# Fecal let-7b and miR-21 directly modulate the intestinal microbiota, driving chronic inflammation

**DOI:** 10.1080/19490976.2024.2394249

**Published:** 2024-09-03

**Authors:** Maite Casado-Bedmar, Maryline Roy, Louis Berthet, Jean-Pierre Hugot, Chunhua Yang, Hana Manceau, Katell Peoc’h, Benoit Chassaing, Didier Merlin, Emilie Viennois

**Affiliations:** a Center for Research on Inflammation, Université Paris Cité, Paris, France; b Department of Pediatric Gastroenterology, Hôpital Robert Debré, Assistance Publique Hôpitaux de Paris (AP-HP), Paris, France; c Institute for Biomedical Sciences, Center for Inflammation, Immunity and Infection, Digestive Disease Research Group, Georgia State University, Atlanta, GA, USA; d Laboratory of Clinical Biochemistry, Beaujon Hospital, APHP, Clichy, France; e Microbiome-Host Interactions, Institut Pasteur, Université Paris Cité, INSERM U1306, Paris, France; f Mucosal Microbiota in Chronic Inflammatory Diseases, INSERM U1016, CNRS UMR8104, Université Paris Cité, Paris, France; g CHRU Nancy, IHU Infiny, Nancy, France; h Veterans Affairs Medical Center, Decatur, GA, USA

**Keywords:** microRNA, gastrointestinal microbiome, dysbiosis, intestinal permeability, colitis, Anti-miR-21, anti-let-7b, host-microbiota interaction, miR-21, let-7b

## Abstract

Inflammatory bowel diseases (IBD) etiology is multifactorial. Luminal microRNAs (miRNAs) have been suspected to play a role in the promotion of chronic inflammation, but the extent to which fecal miRNAs are interacting with the intestinal ecosystem in a way that contribute to diseases, including IBD, remains unknown. Here, fecal let-7b and miR-21 were found elevated, associated with inflammation, and correlating with multiple bacteria in IBD patients and IL-10^–/–^ mice, model of spontaneous colitis. Using an *in vitro* microbiota modeling system, we revealed that these two miRNAs can directly modify the composition and function of complex human microbiota, increasing their proinflammatory potential. *In vivo* investigations revealed that luminal increase of let-7b drastically alters the intestinal microbiota and enhances macrophages’ associated proinflammatory cytokines (TNF, IL-6, and IL-1β). Such proinflammatory effects are resilient and dependent on the bacterial presence. Moreover, we identified that besides impairing the intestinal barrier function, miR-21 increases myeloperoxidase and antimicrobial peptides secretion, causing intestinal dysbiosis. More importantly, *in vivo* inhibition of let-7b and miR-21 with anti-miRNAs significantly improved the intestinal mucosal barrier function and promoted a healthier host-microbiota interaction in the intestinal lining, which altogether conferred protection against colitis. In summary, we provide evidence of the functional significance of fecal miRNAs in host-microbiota communication, highlighting their therapeutic potential in intestinal inflammation and dysbiosis-related conditions, such as IBD.

## Introduction

Inflammatory bowel diseases (IBD) are characterized by dysbiosis and chronic inflammation of the gastrointestinal tract. IBD’s etiology is multifactorial and strongly associated with an imbalance of the gut microbial community. Indeed, studies with IBD patients and mouse models have consistently supported dysbiosis as a key event in the pathogenesis of inflammatory disorders.^1–5^ Recent research identified numerous microRNAs (miRNAs) in feces,^6–8^ suggesting they could play a role in host-microbiota interaction. MiRNAs are small noncoding RNAs that regulate several biological processes by binding messenger RNAs (mRNAs), inducing post-transcriptional gene expression regulation. Remarkably, miRNAs are highly resistant to RNase degradation and can be detected in different biofluids. In IBD patients, miRNAs have been reported deregulated in serum,^9^ mucosa, and stool samples.^10–12^

In the gastrointestinal tract, miRNAs are secreted by the enterocytes into the lumen and accumulate in feces.^13^ Although some studies have found them to be relevant in the maintenance of intestinal homeostasis,^14^ the mechanisms through which microbiota and fecal miRNAs are together involved in intestinal inflammation remain unknown.^15–18^ The main goal of this study was to investigate the pathogenic relevance of miRNA-microbiota interactions in relation to colitis.

We focused on fecal let-7b and miR-21, identified here as key molecules associated with human and murine colitis. While let-7b has yet to be explored in the context of IBD, miR-21 has previously been associated with colon cancer, inflammation, and barrier impairment, without further knowledge of its interactions with bacteria. Herein, we report for the first-time different mechanisms of action of let-7b and miR-21 that ultimately induced intestinal inflammation and microbiota alterations. Furthermore, we explored the therapeutic potential of oral anti-miRNA treatment, which proved to ameliorate colitis, improve intestinal permeability, and prevent dysbiosis. Overall, this study establishes new roles for fecal miRNAs as inter-kingdom mediators that potentially shape the microbiota in detrimental ways to ultimately impact intestinal homeostasis.

## Materials and methods

### Human samples

Fecal human specimens were obtained from 18 healthy controls without gastrointestinal symptoms and 18 IBD patients who presented active intestinal inflammation and fecal calprotectin levels >250 μg/g (Table 1). Participants, age and gender-matched, were recruited from the Laboratory of Clinical Biochemistry at Beaujon Hospital, Paris, France as part of the MICIMUM study. Exclusion criteria for both groups included the use of antibiotics in the last three months, previous colorectal exeresis or intestinal resection within the past 6 months, and use of non-steroidal anti-inflammatory drugs. Fecal samples were stored in anaerobic conditions at −80°C until further use.

**Table 1.** Demographic characteristics of the 18 healthy controls and 18 inflammatory bowel disease (IBD) patients, both ulcerative colitis (UC) and Crohn's disease (CD), included in the study.

	Age Year mean ± SEM	Gender Female n (%)	Disease n (%)	Calprotectin [µg/g] Mean ± SEM
Healthy Control n = 18	37.8 ± 2	12 (66.5%)	–	8.8 ± 3
Active IBD n = 18	37.3 ± 2	12 (66.5%)	UC *n* = 7 (38.9%)	591.7 ± 83
			CD *n* = 10 (55.5%)	
			Undetermined = 1 (5.5%)	

### Mice experiments

#### Screening of fecal miRNAs in IL-10^–/–^ mice

For the initial screening experiment, we used 6 female Interleukin-10 knock-out (IL-10^–/–^) mice, known to develop more severe spontaneous colitis than males.^19^ The 4 weeks-old IL-10^–/–^ mice (B6.129P2-Il10^tm^[1]^Cgn^/J, Strain #:002251, Jackson Laboratory, Bar Harbor, USA) were housed under Specific pathogen-free (SPF) conditions at Georgia State University (USA) and kept on for 13 weeks. Fecal samples were collected at age 37 and 115 days old, respectively, before and during colitis. A total of 752 miRNAs were screened using high-throughput qPCR-based technology.

#### Induction of colitis with miRNAs to C57Bl/6 mice

The 6-week-old wild-type (WT) C57Bl/6 female mice (Charles River, France) were exposed to water (control group, *n* = 21), hsa-miR-21a-5p (miR-21, *n* = 18), or hsa-let-7b-5p (let-7b, *n* = 18) (miRCURY LNA miRNA mimic, Qiagen) diluted in the drinking water at the previously reported dose of 200 nM,^13^ during 4 days (Figure 3(a)). Notice that the possible unspecific effects of our selected miRNA mimics were tested in an additional group of mice (*n* = 6) treated with negative control miRNA [200 nM] (Negative Control miRCURY LNA miRNA Mimic Ref. YM00479902, Qiagen) for 4 days (Supplementary Figure S2).

#### Post-treatment study after induction of colitis with miRNAs to C57Bl/6 mice

To study the inflammation and microbiota evolution after miRNA-induced changes *in vivo*, a set of mice was treated as previously (*n* = 6 per group). After the 4-day administration of miRNAs [200 nM] as described above, drinking bottles were replaced with fresh water, and mice were kept for another 17 days for a post-treatment phase (Figure 6(a)).

#### Induction of colitis with miRNAs to C57Bl/6 mice with depleted microbiota

To study if the miRNAs’ inflammatory potential was dependent on the microbiota, Bacteria were depleted by using an antibiotic cocktail diluted in the drinking water [Ampicillin 1 mg/mL (Sigma) and Neomycin 0.5 mg/mL, (Sigma)] for 14 days prior the 4-days miRNAs administration (*n* = 9 per group, Figure 6(i)).

#### Germ-free experiment

Germ-free C57Bl/6 mice (GF) were kept under germ-free conditions in a Park Bioservices isolator in the gnotobiotic facility of Cochin Institute, Paris (France). Six-week-old wild-type C57Bl/6 GF female mice were exposed to water (control group, *n* = 4), miR-21 [200 nM] (*n* = 4), or let-7b [200 nM] (*n* = 4) diluted in the drinking water for 4 days, as described above.

### Long-term therapeutic administration of anti-miRNAs in IL-10^–/–^ mice

In-house bred IL-10^–/–^ 4–5 weeks-old mice (day 0) were kept until 14–15 weeks of age. Mice were gavaged from day 0 with either 150 μl of PBS (control group, *n* = 9), mmu-mir-21a-5p microRNA inhibitor (anti-miR-21, *n* = 9) [0.1 nmol], or mmu-let-7b-5p microRNA inhibitor (anti-let-7b, *n* = 9) [0.1 nmol] (miRCURY LNA miRNA Power Inhibitors, Qiagen) 3 times per week. After 10 consecutive weeks of treatment (day 71), mice were euthanized (Figure 7(a)).

#### Short-term therapeutic administration of anti-miRNAs in DSS-induced colitis in C57Bl/6 mice

Acute colitis was induced to a total of 24 C57Bl/6 female mice with 1% Dextran Sulfate Sodium (DSS) diluted in the drinking water from day 0 to 7. Mice were gavaged daily from day 1 with either 150 μl of PBS (negative control group, *n* = 6), 1% DSS (positive control group, *n* = 6), anti-miR-21 diluted in 1% DSS [0.1 nmol] (*n* = 6), or anti-let-7b diluted in 1% DSS [0.1 nmol] (*n* = 6) (miRCURY LNA miRNA Power Inhibitors, Qiagen). Mice were euthanized on day 7.

Body weight and fresh fecal samples were collected regularly. Macroscopic evidence of inflammation was evaluated by measuring colon length, colon weight, and spleen weight. Blood and colonic samples were collected for further analysis.

### Ussing chambers assay

Intestinal permeability was assessed in Ussing chambers as previously described.^20^ Briefly, proximal colonic samples were opened along the mesenteric border and mounted in Ussing chambers (Easy Mount P2312; Physiologic Instrument). Mounted samples were equilibrated for 20 min in 4 ml of 10 mM glucose (Sigma, pH 7.4) on the serosal side and 4 ml of 10 mM mannitol (Sigma, pH 7.4) on the mucosal side. Chambers were kept at a constant temperature of 37°C and gassed with 95% O_2_/5% CO_2_. The permeability assay started when adding 1 mg/mL of the paracellular probe FITC-Dx 4 kDa (TdB Labs AB) and 10^−5^ M of the transcellular marker horseradish peroxidase 45 kDa (HRP, Type VI, Sigma).^21^ Serosal samples were collected at 0, 30, 60, 90, and 120 min. FITC-Dx 4 kDa passage was measured at 488 nm in a SPARK 10 M plate reader. HRP was measured using the QuantaBlu™ Fluorogenic Peroxidase Substrate Kit (Pierce) according to a previously described protocol.^22^ The passage of both markers was calculated using standard curves and expressed as ∆120-30 min.

### Serum immunoreactivity to LPS by ELISA

Serum immunoreactivity to lipopolysaccharide (LPS) was examined by ELISA as previously described.^23^ High-binding ELISA plates were coated overnight with purified LPS from *E. Coli* 0128:B12 (2 μg/well, Sigma) in a 9.6 pH bicarbonate buffer. Pre-diluted sera (1:200) were added to the wells and incubated at 37°C for 1 h. Wells were washed and incubated with HRP-conjugated anti-mouse IgG (1:1,000, SAB3701095, Sigma) at 37°C for 1 h. After washing, the peroxidase substrate tetramethylbenzidine (TMB, Sigma) was added and stopped after 15 min incubation with stop solution (2N H_2_SO_4_). Plates were then read at 450 nm with wavelength correction set at 540 nm in a SPARK 10 M plate reader (Tecan). Data are reported as percentages to control.

### Staining of colonic tissue and histopathologic analysis

Mouse proximal colons were fixed in 4% formaldehyde, dehydrated, and embedded in paraffin blocks. Tissues were sectioned at 4 μm thickness and stained with hematoxylin and eosin (H&E) using standard protocols. Images were acquired using a Lamina Slide Scanner (Perkin Elmer) at the Hist’IM platform (INSERM U1016, Paris, France). The H&E stained samples were blindly scored to study the mucosa status and colitis level, as previously described.^24^ The score was based on the infiltration of inflammatory cells as follows: (0) no cell infiltration, (1) light infiltrate in the lamina propria, (2) small lymphoid follicle 500 μm^2^ − 2,000 μm^2^, (3) medium lymphoid follicle 2,000 μm^2^ − 30,000 μm^2^, (4) large lymphoid follicle >30,000 μm^2^.

### Colonic myeloperoxidase assay

The activity of myeloperoxidase (MPO), a neutrophil enzyme and marker of inflammation, was analyzed as previously described.^25^ Briefly, distal colonic tissues were mechanically homogenized at 50 mg/ml in 0.5% hexadecyltrimethylammonium bromide (Sigma) diluted in 50 mM PBS (pH 6.0), sonicated, freeze-thawed 3 times, and centrifuged for 15 min at 14,000 rpm 4°C. The collected supernatant (50 μL) was mixed with freshly prepared Reactive Buffer (200 μL) consisting of 1 mg/mL of dianisidine dihydrochloride (Sigma) and 5 × 10^−4^% H_2_O_2_ (Sigma). The change in optical density was measured at 450 nm in a SPARK 10 M plate reader (Tecan). Human neutrophil MPO (Sigma) was used to create the standard curve.

### Preparation of fecal supernatant

Fecal samples were reconstituted in PBS to a final concentration of 100 mg/mL, vortexed for 15 min, and centrifuged for 10 min at 14,000 g 4°C. Fecal supernatant was collected, serially diluted, and stored at −80°C until further use.

### Quantification of fecal lipocalin-2 by ELISA

Lipocalin-2 was measured in previously processed fecal supernatant following the manufacturer’s instructions of the murine Lipocalin-2/NGAL ELISA kit (R&D Systems). Optical density was read at 450 nm SPARK 10 M plate reader (Tecan). Samples were normalized per group to Day 0 for each experiment.

### Colonic mRNA isolation and quantitative real-time qPCR assays

Colonic tissues were collected during euthanasia, placed in RNA-later (Invitrogen), and kept at −80°C until further use. NucleoSpin RNA kit (Macherey-Nagel™) was used to isolate the total mRNA from colonic samples according to the manufacturer’s instructions. Extracted mRNAs were quantified using NanoDrop One Spectrophotometer (Ozyme). After cDNA synthesis, inflammatory cytokines were measured by RT-qPCR with TaqMan using a LightCycler 96 system (Roche). The mRNA expression levels of tight junction proteins, mucus, and defensins were measured by RT-qPCR with SYBR Green (Qiagen). Changes in mRNA expression were determined by calculating the fold changes using the comparative threshold cycle (Ct) method normalizing to hsa-miR-194-5p expression, chosen for its stability index in both colitic mice (0.3822) and human IBD (0.03629).

### MiniBioReactor Array assay

The MiniBioReactor Array (MBRA) assay is a novel and cost-effective system designed for high-throughput and reproducible cultivation of fecal bacterial communities for up to 3 weeks. This system is particularly valuable for studying various aspects of the intestinal microbiota, including the recent study of dietary emulsifier effects on gut inflammation.^26^ Pioneering, here we explored the direct impact of miR-21 and let-7b on complex human microbiota in MBRA. Briefly, and as previously described by Naimi et al.,^26^ the MBRA system was placed in an anaerobic chamber at 37°C. A total of 18 independent chambers containing 15 mL of BRM culture medium were maintained by two peristaltic pumps adapted for low flow rates. We followed Auchtung et al.’s protocol^27^ for sample preparation and fecal inoculation into 9 chambers for each healthy donor. After a resting time of 24 h, the flow rate was set to 1.9 mL/h (equivalent to an 8 h turnover, mimicking the physiological intestinal peristalsis). After 120 h of stabilization, PBS, miR-21, or let-7b (miRCURY LNA miRNA mimic, Qiagen) were injected at a final concentration of 200 nM. In order to ensure the interaction between miRNAs and microbiota, a second injection of the treatments was administered after 12 hours. Sample collections were performed at different time points, as indicated in Supplementary Figure S1c.

### Fecal and colonic miRNA extraction and quantification

Small RNA molecules (<200 nt) were obtained from the colonic and fecal samples collected using the mirVana isolation kit (Thermo Fisher) according to the manufacturer’s instructions. Briefly, samples were disrupted and homogenized in Lysis buffer using TissueLyser (Qiagen). The homogenate was mixed with chloroform and centrifuged. The aqueous phase was mixed with 1/3 volumes of ethanol and loaded into a RNeasy spin column. The collected filtrate was mixed with 2/3 volumes of ethanol and filtered for a second time. After 3 washes, small RNAs were eluted with RNase-free water. The quality and quantity of small RNAs were analyzed using NanoDrop One Spectrophotometer (Ozyme). The miRCURY LNA RT kit (Qiagen) was used for the cDNA synthesis from the pre-diluted small RNA samples (10 ng/μl). Obtained cDNA was mixed with the 2X miRCURY SYBR Green Master Mix (Qiagen) and RT-qPCR was performed in a LightCycler 96 system (Roche) using hsa-miR-21-5p and hsa-let-7b-5p. The UniSp6 RNA spike-in was used as an interpolate calibrator. Changes in miRNA expression were determined by calculating the fold changes using the comparative Ct method with a normalization with hsa-miR-194-5p, chosen for its stability index in colitic mice.

### Quantification of fecal LPS and flagellin load

Fecal load of LPS and flagellin were quantified using HEK-Blue-mTLR4 and HEK- Blue-mTL5 cells, respectively (Invivogen), as in precedent reports.^28,29^ Previously processed fecal supernatant was applied to the mammalian cells and incubated for 24 h at 37°C. Cell culture supernatants were applied to QUANTI-Blue medium (Invivogen) and alkaline phosphatase activity was measured at 620 nm after 30 min. Purified LPS from *Escherichia coli* (Sigma) and flagellin from *Salmonella typhimurium* (Sigma) were used for standard curve determination. Optical density was read at 450 nm in a SPARK 10 M plate reader (Tecan).

### Bacterial quantification

For quantification of the total fecal bacterial load, total bacterial DNA was isolated from weighted feces using QIAamp DNA Stool Mini Kit (Qiagen) after a step of mechanical disruption (bead beating). Total DNA yields were measured using a nanodrop.

### Localization of bacteria by FISH immunostaining

To analyze bacteria localization at the surface of the intestinal mucosa, colonic tissues were analyzed after mucus immunostaining paired with fluorescent *in situ* hybridization (FISH), as previously described.^30^ Briefly, proximal colonic tissues were placed in Carnoy’s fixative solution and stored at 4°C until further use. Samples were washed in methanol (2 × 30 min), absolute ethanol (2 × 15 min), 50% ethanol/50% xylene (15 min), and xylene (2 × 15 min). Tissues were then embedded in paraffin blocks and cut in 4 μm. Sections were dewaxed with xylene (2 × 10 min) at 60°C and ethanol 99.5% (5 min). Slides were then incubated overnight at 50°C together with the hybridization solution (20 mM Tris – HCl, pH 7.4, 0.9 M NaCl, 0.1% SDS, 20% formamide) containing 10 mg/ml of EUB338 probe (50-GCTGCCTCCCGTAGGAGT-30, labeled with Alexa 647). Slides were washed (10 min) with washing buffer (20 mM Tris – HCl, pH 7.4, 0.9 M NaCl), quickly with PBS, and then incubated with blocking solution (5% fetal bovine serum in PBS) for 30 min at 4°C. Rabbit mucin-2 primary antibody (1:100, GTX100664, Genetex) was incubated overnight at 4°C. After washing in PBS (3 × 10 min), secondary anti-rabbit Alexa 488 (1:200, Sigma) was diluted together with Phalloidin-TRITC (100 mg/mL, Sigma) and Hoechst 33,258 (10 mg/ml, Sigma) and incubated at room temperature for 2 h. Slides were washed (3 × 10 min) in PBS and mounted using Prolong anti-fade mounting media. An average of 5 images were collected per slide, in duplicates, with LSM 780 (ZEISS) in a 20× objective and processed with ImageJ Fiji. The distance of the 25 closest bacteria to the intestinal epithelial layer was measured per image. The average of all the images per slide was calculated per mouse and represented as the average distance of bacteria to the epithelium (μm).

### Microbiota analysis by 16S rRNA gene sequencing using illumina technology

Microbiota analyses were performed before and after treatment as indicated in each experiment. 16S rRNA gene amplification and sequencing were done using the Illumina MiSeq technology following the protocol of Earth Microbiome Project with their modifications to the MOBIO PowerSoil DNA Isolation Kit procedure for extracting DNA (https://press.igsb.anl.gov/earthmicrobiome). Bulk DNA was extracted from frozen feces using DNeasy® 96 PowerSoil® Pro QIAcube® HT Kit (Qiagen) with mechanical disruption (bead-beating). The 16S rRNA genes, region V4, were PCR amplified from each sample using a composite forward primer and a reverse primer containing a unique 12-base barcode, designed using the Golay error-correcting scheme, which was used to tag PCR products from respective samples). We used the forward primer 515F *5’-AATGATACGGCGACCACCGAGATCTACACGCTXXXXXXXXXXXX***TATGGTAATT*GT***GTGYCAGCMGCCGCGGTAA*-3*’: the italicized sequence is the 5’ Illumina adaptor, the 12 X sequence is the Golay barcode, the bold sequence is the primer pad, the italicized and bold sequence is the primer linker, and the underlined sequence is the conserved bacterial primer 515F. The reverse primer 806 R used was *5’-CAAGCAGAAGACGGCATACGAGAT***AGTCAGCCAG*CC***GGACTACNVGGGTWTCTAAT*-3’*: the italicized sequence is the 3’ reverse complement sequence of Illumina adaptor, the bold sequence is the primer pad, the italicized and bold sequence is the primer linker and the underlined sequence is the conserved bacterial primer 806 R. PCR reactions consisted of Hot Master PCR mix (Quantabio), 0.2 mM of each primer, 10–100 ng template, and reaction conditions were 3 min at 95°C, followed by 30 cycles of 45 s at 95°C, 60 s at 50°C and 90 s at 72°C on a Biorad thermocycler. PCR products were quantified with the Quant-iT PicoGreen dsDNA assay. Then, a master DNA pool was generated from the purified products in equimolar ratios and purified with Ampure magnetic purification beads (Agencourt). The pooled product was quantified using the Quant-iT PicoGreen dsDNA assay and then sequenced using an Illumina MiSeq sequencer (paired-end reads, 2 × 250 bp) at the Genom’IC sequencing facility of Cochin Institute. 16S rRNA sequences were then analyzed using QIIME2—version 2019.51 Sequences were demultiplexed and quality filtered using the DADA2 method with QIIME2 default parameters in order to detect and correct Illumina amplicon sequence data, and a table of QIIME2 artifact was generated. A tree was next generated, using the align-to-tree-mafft-fasttree command, for phylogenetic diversity analyses, and alpha and beta diversity analyses were computed using the core-metrics-phylogenetic command. PCoA plots were used to assess the variation between the experimental group (beta diversity) and analyzed with PERMANOVA. For the taxonomic analyses, features were assigned to operational taxonomic units (OTUs) with a 99% threshold of pairwise identity to the Greengenes reference database.^31^

### Statistical analysis

Data are expressed as mean ± SEM and statistical analyses were performed using GraphPad Prism software (V.8). After testing for normality, significance was determined using unpaired t-tests or the Mann – Whitney U test when comparing two groups, and one-way ANOVA or Kruskal-Wallis when comparing more than two groups with a Bonferroni post hoc test. For data collected at different time points, a two-way repeated-measures ANOVA or a mixed-effects model (if missing values) with a Bonferroni post hoc test. For the high-throughput qPCR-based technology, only miRNAs with at least 3 values per group were included in the analysis. After the average of the normalized Cp values was calculated, t-student tests followed by a Benjamini-Hochberg procedure were performed. Multivariable Associations with Linear Models (MaAsLin 2) of the microbial features were performed with the MaAsLin2 R package.^32^ Nominal p-values across all associations were then adjusted using the Benjamini – Hochberg FDR method. An FDR <0.25 is the standard default using MaAsLin2. Significances were noted as indicated in the figure legends. Results were considered significant at *p* < 0.05.

## Results

### Fecal let-7b and miR-21 are associated with colonic inflammation in humans and mice

**Figure 1.**
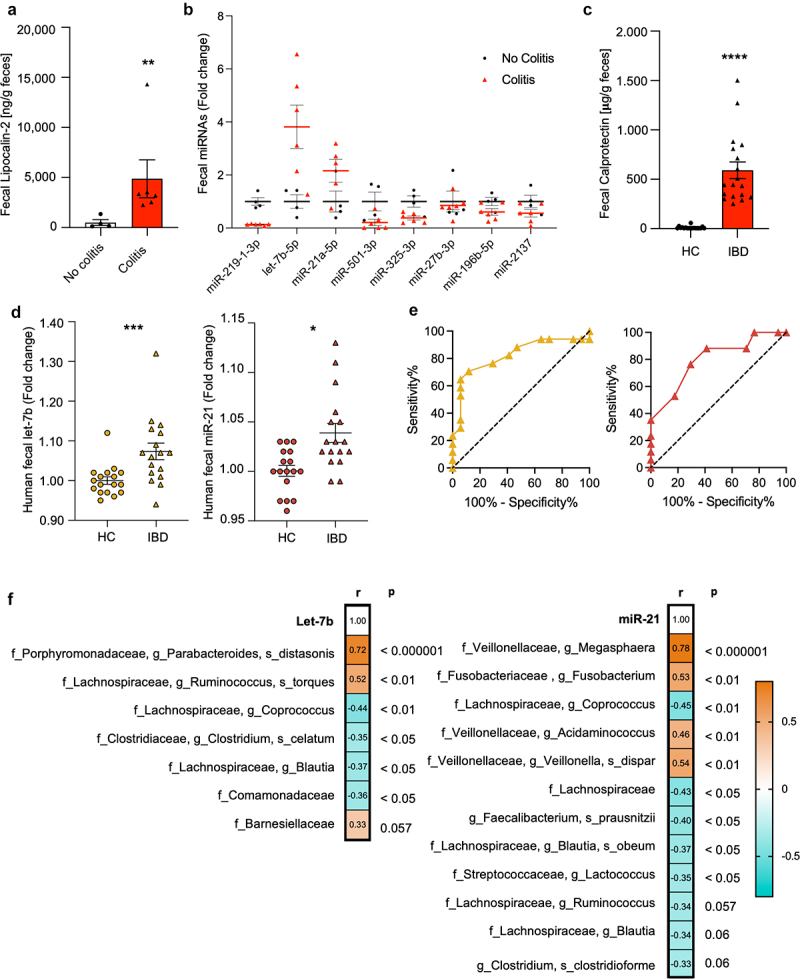
Let-7b and miR-21 are inflammation-associated miRNAs in mice and IBD patients.

We first used IL-10^–/–^ mice, known to spontaneously develop chronic enterocolitis,^5^ to identify miRNAs associated with intestinal inflammation. Fecal miRNAs of host origin were analyzed before and during colitis as attested by levels of fecal lipocalin-2, a dynamic marker of intestinal inflammation (Figure 1(a)). A total of 752 miRNAs were screened using high-throughput qPCR-based technology, with the 8 most altered miRNAs being presented in Figure 1(b). Among those, the 2 most increased fecal miRNAs were let-7b-5p (let-7b) and miR-21a-5p (miR-21).

(a-b) Fecal samples were collected and analyzed from IL-10^–/–^ mice (*n* = 6) before and after the development of enterocolitis. (a) Levels of fecal lipocalin-2, marker of intestinal inflammation. (b) Expression profiles of the 8 most deregulated miRNAs in IL-10^–/–^ mice before (black dots) and after (red triangles) colitis. The average expression of each miRNA before colitis was set to 1. After the average of the normalized Cp values were calculated, t-student tests followed by a Benjamini-Hochberg procedure were performed. (c-e) Fecal samples from a cohort of 18 healthy controls (HC) and 18 inflammatory bowel disease patients (IBD) were collected. (c) Fecal levels of the proinflammatory marker calprotectin, (d) miR-21 and let-7b. (e) ROC curves showing miR-21 (AUC 0.80, *p* < 0.01) and let-7b (AUC 0.82, *p* < 0.01) discriminating between IBD and HC. (f) Heat-maps showing bacterial taxa (in rows) that were significantly correlated with let-7b and miR-21 (in columns) using Multivariable Associations with Linear Models (MaAsLin 2) and the Benjamini – Hochberg FDR method. Orange and blue indicate positive and negative correlations, respectively. For both bar and dot plots, values are given as mean ± SEM and significant difference is recorded as *p < 0.05, **p < 0.01, ***p < 0.001, ****p < 0.0001.

To assess their clinical relevance in the pathogenesis of human colitis, we analyzed let-7b and miR-21 in a cohort of 18 IBD and 18 healthy controls matched by age and gender. The available demographics are summarized in Table 1. Levels of fecal calprotectin confirmed the presence of inflammation in IBD and its absence in healthy controls (Figure 1(c)). Consistently, the analysis by RT-qPCR demonstrated an upregulation of fecal let-7b and miR-21 in patients (Figure 1(d)), which positively correlated with calprotectin concentrations (let-7b: *r* = 0.477, *p* < 0.01; miR-21: *r* = 0.381, *p* < 0.05). These findings indicate a potential association between these miRNAs and intestinal inflammation in humans. Further, the analysis of receiver operating characteristic (ROC) curves showed let-7b (AUC 0.82, *p* < 0.01) and miR-21 (AUC 0.80, *p* < 0.01) to remarkably discriminate between IBD and healthy controls with high sensitivity and specificity (Figure 1(e)). These results suggest that fecal let-7b and miR-21 may also serve as valuable diagnostic markers of colitis.

### Fecal miRNAs correlate with human gut microbiota

To investigate the relationship between the microbiome and the identified proinflammatory miRNAs, we performed a 16S rRNA sequencing analysis from human stool samples. Our results confirmed the presence of classic IBD dysbiotic features with decreased alpha diversity (Supplementary Figure S1a,b), matching the current understanding of an altered composition in patients.^2^ Further, correlation analyses showed that fecal levels of let-7b and miR-21 significantly correlated with different bacteria (Figure 1(f)). Results indicated a strong let-7b correlation, among others, with *Parabacteroides distasonis* which has recently been implicated with CD.^33^ In contrast, miR-21 showed a strong and positive correlation with the genus *Megasphaera*, known to synthesize short-chain fatty acids,^34^ and negative correlation with the anti-inflammatory commensal bacterium *Faecalibacterium prausnitzii*.^35^ A consistent pattern was found between miR-21 and its associations with bacterial families. Interestingly, miR-21 mostly exhibited negative correlations with bacterial species within the *Lachnospiraceae* family and positive with species from the *Veillonellaceae* family (Figure 1(f)). These results suggest possible direct interactions between fecal let-7b and miR-21 and the microbiota, each with distinct specificities.

### The colitis-associated miRNAs let-7b and miR-21 can directly alter human complex microbiota in MBRA systems

**Figure 2.**
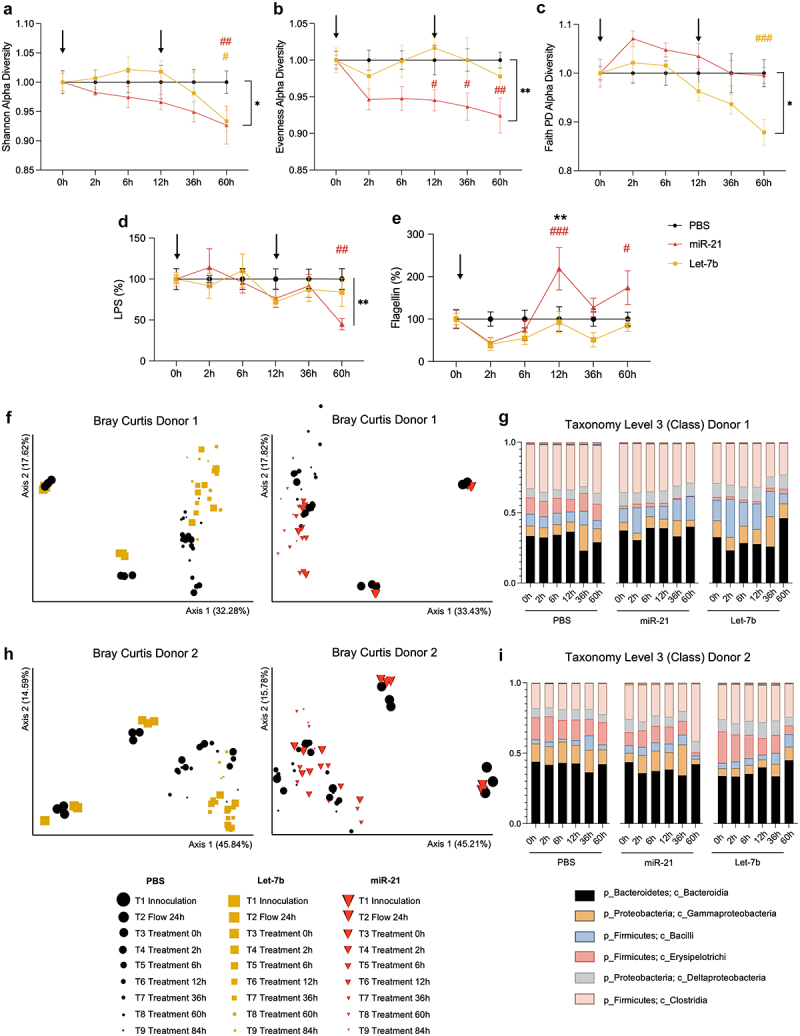
Let-7b and miR-21 directly affect human microbiota composition.

To elucidate direct interactions of let-7b and miR-21 with bacteria, we next examined their impact on a complex human microbiota cultured *in vitro* in a MiniBioReactor Array (MBRA) system.^27,36^ Human fecal samples from 2 healthy donors were used to generate anaerobic complex cultures that were incubated with either PBS, let-7b, or miR-21 for a total of 60 h (Supplementary Figure S1c). The analysis of the 16S rRNA gene sequences showed that both miRNAs reduced the richness of the microbial population (Shannon, Figure 2(a)). Interestingly, miR-21 had a severe effect on the species distribution of abundance (Evenness, Figure 2(b)), while let-7b drastically diminished the phylogenetic diversity (Faith PD, Figure 2(c)). Shannon and Evenness are abundance-based alpha diversity indexes, extremely sensitive to the singleton count, whereas Faith’s PD does not consider species abundances but the presence or absence of a species. Hence, these results indicated that miR-21 may target more abundant bacteria while let-7b affects less abundant species.

Inoculated fecal samples from 2 healthy human donors were incubated with either PBS, miR-21 [200 nM], or let-7b [200 nM] for 60 h in an MiniBioReactor Arrays (MBRA) system. To ensure the presence of miRNAs, shots were administered at times 0 and 12 h (indicated with arrows). (a) Shannon, (b) Evenness, and (c) Faith PD alpha diversity indexes were represented over time and normalized to the control group (PBS). (d) Lipopolysaccharide (LPS) and (e) flagellin measurements. Two-way ANOVA followed by a Bonferroni post hoc test and significant differences were recorded as *p < 0.05, **p < 0.01, when compared to PBS; and #p < 0.05, ##p < 0.01, ###p < 0.001, when compared to time 0 h. (f and h) PCoA of Bray Curtis from donor 1 (f) and 2 (h) with all-time points. Dots represent PBS-treated samples, inverted triangles are miR-21, and squares let-7b treated samples. Symbol size is proportional to the time of the experiment, the smaller the later in the experiment. (g and i) Taxonomic summary at the class level from donors 1 (g) and 2 (i).

The measurement of bioactive LPS and flagellin was assessed to identify the proinflammatory potential of the microbiota.^29^ Both mimics reduced LPS over time, but only the miR-21 group reached significance (Figure 2(d)). In contrast, *in vitro* flagellin levels increased after miR-21 incubation, but not let-7b (Figure 2(e)).

Using Principal coordinates analysis (PCoA), we found that samples incubated with miRNAs were well clustered and distinguished from those treated with PBS (let-7b *vs*. PBS donor 1 q < 0.001, donor 2 q < 0.005; miR-21 *vs*. PBS donor 1 q < 0.001, donor 2 q < 0.05; Figures 2(f,h)). The taxonomic display over time from each donor revealed a stable microbiota in the PBS groups, whereas when treated with miRNAs, some classes of bacteria, such as *Bacteroidetes Bacteroidia*, known to be decreased in Crohn’s Disease (CD) patients,^37^ were notably affected by both let-7b and miR-21 (Figures 2(g,i)). In order to validate the previously observed associations between the miRNAs and certain bacteria (Figure 1(f)), the relative abundance of each microorganism was further examined. Analysis revealed a significant decrease of *Clostridia Clostridiales Lachnospiraceae Blautia* and *Ruminococcus* at class and family levels in those human samples incubated with either let-7b and miR-21 (Supplementary Figure S1d). Meanwhile, the remaining correlating bacteria did not exhibit significant differences between groups (data not shown).

Altogether, our findings suggest that let-7b and miR-21 directly interact with bacteria and reshape the microbiota. Importantly, these miRNA-bacteria interactions are miRNA-specific and likely to have functional implications, particularly in modifying the microbiota pro-inflammatory potential. To determine the importance of these molecules in the pathophysiology of colitis, the effects of let-7b and miR-2 were further analyzed *in vivo* in different mouse models.

### Exogenous administration of let-7b and miR-21 drives miRNA-specific intestinal inflammation

**Figure 3.**
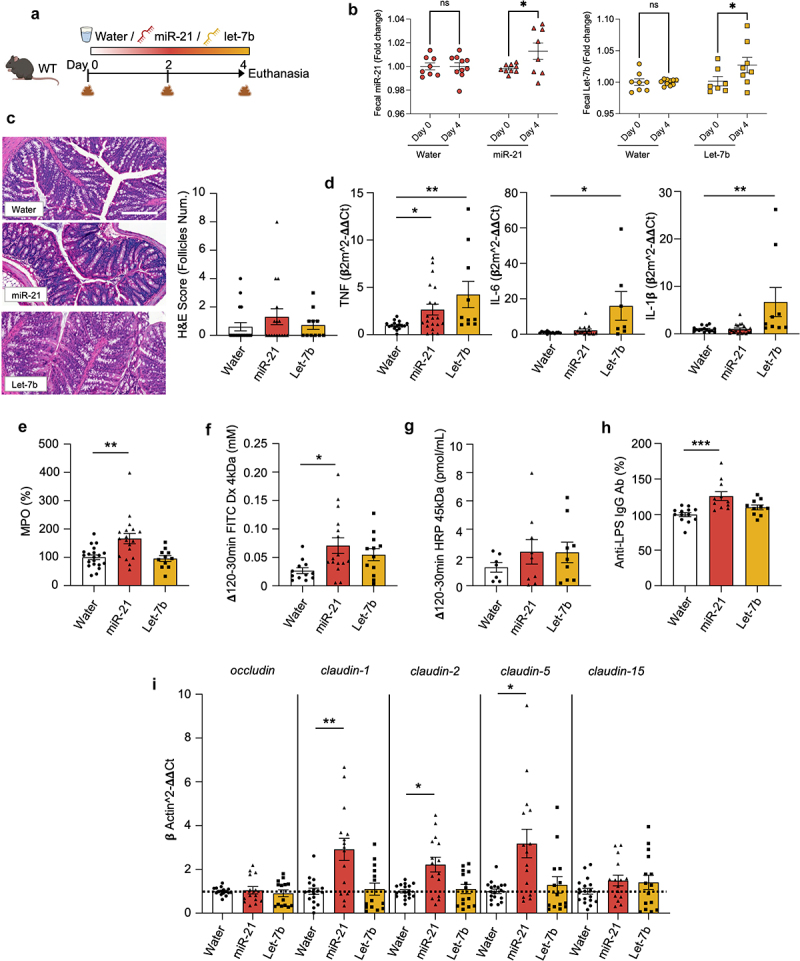
Administration of exogenous miRNAs induced colonic inflammation, but only miR-21 treatment impaired the intestinal barrier function.

Having established the association between let-7b and miR-21 with intestinal inflammation, we next determined to which extent they are central in driving the inflammatory response *in vivo*. WT mice were treated *ad libitum* in the drinking water for 4 days with either water, let-7b, or miR-21 (Figure 3(a)). The presence of these exogenously administered molecules was evaluated throughout the entire experiment, confirmed stable in the water (Supplementary Figure S2a), and significantly increased in the feces of administered mice, indicating miRNAs reached the colonic lumen successfully (Figure 3(b)).

(a) C57Bl/6 wild-type (WT) mice were treated *ad libitum* with either water (*n* = 21), miR-21 [200 nM] (*n* = 18), or let-7b [200 nM] (*n* = 18) for 4 days. (b) Fecal miRNAs relative amounts at day 0 and 4 in the water (circles), miR-21 (triangle), and let-7b groups (square). (c) Microscopic inflammation studied in the colon of WT mice after staining with H&E and histological scored. Bar = 200 μm. (d) Measurement of relative amounts of colonic mRNA expressions levels of the proinflammatory cytokines TNF, IL-6, and IL-1β at day 4. (e) Colonic amounts of the neutrophil inflammatory marker myeloperoxidase (MPO). (f-g) Intestinal permeability was studied *ex vivo* by quantifying the (f) paracellular and (g) transcellular flow of FITC-Dx 4 kDa and HRP 45 kDa over 120 min. (h) Serum was collected at euthanasia and anti-lipopolysaccharide (anti-LPS) IgG antibodies were measured. (i) The mRNA expression of tight junctions were measured by RT-qPCR. Data are represented as mean ± SEM. Statistical analyses were performed using one-way ANOVA followed by a Bonferroni post hoc tests or Kruskal-Wallis followed by Dunn’s post hoc test. Significant differences were recorded as *p < 0.05, **p < 0.01, ***p < 0.001.

As observed by histological examination and quantified by hematoxylin and eosin (H&E) scoring (Figure 3(c)), miRNA-treated mice did not display severe inflammation. Nonetheless, low-grade colitis was detected when quantifying the gene expression of proinflammatory cytokines (Figure 3(d)). The mRNA levels of tumor necrosis factor (TNF) were significantly increased after the treatment with both let-7b and miR-21. Interestingly, we observed a miRNA-dependent inflammatory response. Elevated luminal levels of let-7b led to an enhanced mRNA expression of interleukin (IL)-6 and IL-1β (Figure 3(d)), known as a mRNA expression signature linked with macrophages^38^ and previously identified as hallmark cytokines in chronically inflamed tissues from patients with active IBD.^39^ On another hand, miR-21 enhanced the levels of the neutrophil marker myeloperoxidase (MPO). MPO is a peroxidase mostly kept in the nitrogenophilic granules of neutrophils and released upon their activation^40^ (Figure 3(e)), commonly found in acute inflammation. Conversely, the administration of negative-control-mimic did not induce any sign of inflammation (Supplementary Figure S2b,c), providing evidence of the specificity of let-7b and miR-21. Consequently, the negative-control-mimic group was no longer included in the subsequent experiments. Altogether, these data revealed that the luminal increase of either let-7b or miR-21 is sufficient to create a low-grade colitis in which the inflammatory pathway triggered is miRNA specific.

### Administration of miR-21 increases colonic permeability and bacterial passage

Intestinal inflammation is usually associated with epithelial barrier impairment, which increases intestinal permeability. Therefore, we next measured the flux of FITC-Dx 4 kDa and HRP 45 kDa in colonic samples mounted in Ussing chamber. We observed a significant increase in the paracellular permeability after miR-21, but not let-7b (Figure 3(f)), and a slight increase in the transcellular permeability after either mimic (Figure 3(g)). Increased intestinal permeability associated with the passage of bacterial components was confirmed by measuring circulating anti-LPS IgG antibodies, which were significantly elevated only in the miR-21 group (Figure 3(h)). We then sought to investigate the degree of barrier dysfunction and analyzed the expression of the most relevant genes involved in tight junctions. Only miR-21 significantly increased the expression of *claudin-1, -2*, and *-5* (Figure 3(i)), as commonly observed in intestinal inflammatory disorders.^41^ These results indicate miR-21 effects upon the intestinal barrier function are remarkably stronger than those of let-7b, and lead to increased permeability and passage of bacterial components.

### Administration of let-7b modifies fecal microbiota composition to a greater extent than miR-21

**Figure 4.**
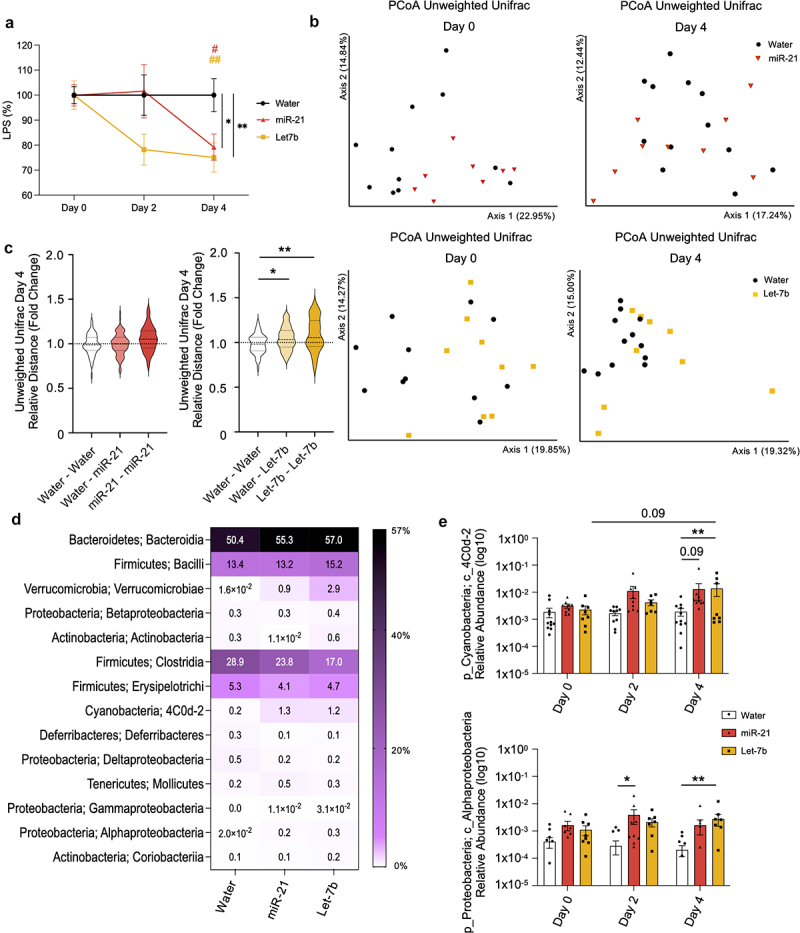
The treatment with miR-21 and let-7b alters the microbiota *in vivo*.

Various methods were used to investigate the effect of enhanced luminal miRNAs on microbiota *in vivo*. Firstly, we investigated the bioactive fecal levels of LPS to evaluate the functional features of the microbiota.^28^ Mice that received mimics showed a drastic decrease in LPS after 2 days of let-7b administration and after 4 days of miR-21 (Figure 4(a)). Gut microbiota was further assessed by 16S rRNA gene sequencing on WT mice stool samples. PCoA of the beta diversity showed that the let-7b-treated group clustered differently than the water-treated groups (Figure 4(b)). Significant modifications of the beta diversity were also confirmed by measuring the relative distance between groups (Figure 4(c)). However, such differences were not detected after miR-21 (Figures 4(b,c)). Similarly, the taxonomic analysis at the class level revealed bacterial differences mostly after let-7b (Figure 4(d)). The two most significantly modified bacterial classes were compared per treatment over time and exhibited a remarkable abundance enhancement of *Cyanobacteria* 4C0d-2 and *Proteobacteria Alphaproteobacteria* in mice treated with mimics compared with the control group (Figure 4(e)). Taken together, this data reveals that luminal miRNA holds the potential to functionally modulate the intestinal microbiota and that let-7b modifies gut microbiota composition *in vivo* more significantly than miR-21.

C57Bl/6 wild-type (WT) mice were treated *ad libitum* with water (*n* = 21), miR-21 [200 nM] (*n* = 18), or let-7b [200 nM] (*n* = 18) for 4 days. (a) Bioactivity of fecal lipopolysaccharide (LPS) was measured using TLR4-HEK cells. Data were represented as mean ± SEM. Two-way ANOVA followed by a Bonferroni post hoc test showed significant differences, recorded as *p < 0.05 and **p < 0.01, when compared to water; and #p < 0.05, ##p < 0.01, when compared to time 0 h. (b) PCoA of the unweighted Unifrac distance matrix of miR-21 (top) and let-7b (bottom) treated mice microbiota at days 0 (left) and 4 (right). (c) Relative distance of treated groups at day 4. Data are represented in violin plots as median ± quartiles. Statistical analyses were performed using a PERMANOVA and Kruskal-Wallis followed by a Dunn’s post hoc test. Significant differences were recorded as *p < 0.05 and **p < 0.01 vs “Water-Water” at D4. (d) Microbiota samples were compared at the class level after treatment and (e) the most significantly modified bacteria were compared overtime. Statistical analyses were performed using a Mixed-effects analysis followed by a Bonferroni post hoc test and significant differences were recorded as *p < 0.05 and **p < 0.01, when compared to water.

### miRNAs impact intestinal mucus homeostasis and promote the production of antibacterial factors

**Figure 5.**
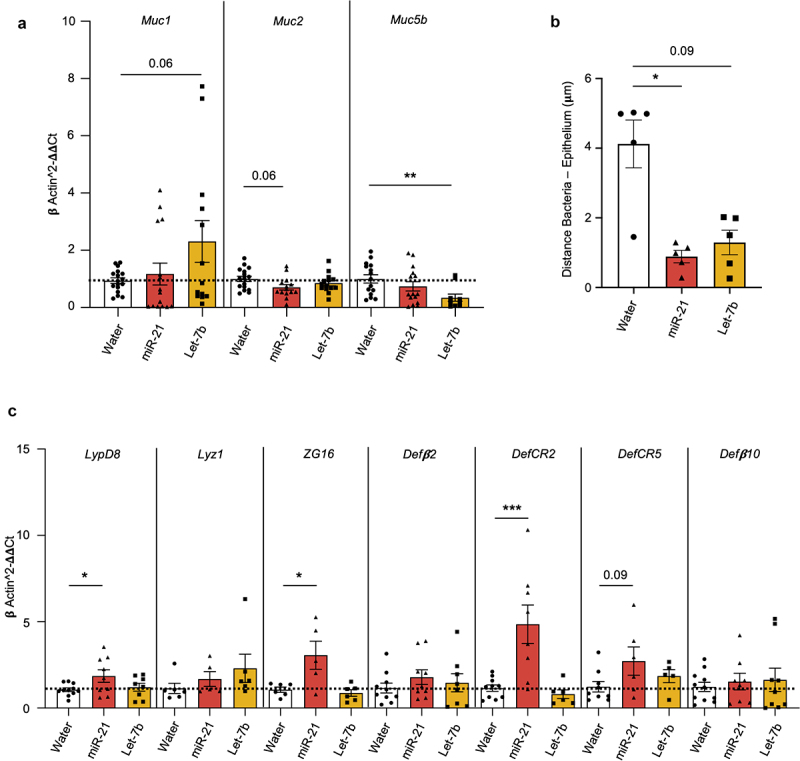
The administration of exogenous miRNAs modified the mucus composition and promoted the production of antibacterial factors.

To determine whether luminal miRNAs may reshape gut microbiota by modifying the bacterial niche, we first analyzed markers of mucus composition by RT-qPCR. After miR-21, the mRNA expression of the most predominant mucin in the colon Mucin 2 (*Muc2*) exhibited a reduction, although this did not reach statistical significance. Whereas, let-7b showed a trend to increase in the expression of *Muc1*, which primary function is cell protection; and significantly decreased *Muc5b*, mainly found in goblet cells (Figure 5(a)). Fecal miRNAs could also induce microbiota to penetrate the mucus, which is associated with the promotion of chronic low-grade intestinal inflammation.^42^ Examination of the microbiota encroachment using confocal imaging of FISH-stained colonic samples revealed that the average bacteria- epithelium distance was reduced after miR-21 and let-7b administration, which was only significant for the first one (Figure 5(b)).

C57Bl/6 wild-type (WT) mice were treated *ad libitum* with either water (*n* = 21), miR-21 [200 nM] (*n* = 18), or let-7b [200 nM] (*n* = 18) for 4 days. (a) mRNA expressions levels of mucus proteins were measured by RT-qPCR in colonic samples. (b) Distances of the closest bacteria to intestinal epithelial cells after treatment per condition over 4 – 6 power fields in 2 different sections per mouse. (c) Antimicrobial peptides expressions were measured by RT-qPCR in colonic samples of mice at day 4. Data are represented as means ± SEM. Statistical analyses were performed using Kruskal-Wallis followed by Dunn’s post hoc tests. Significant differences were recorded as *p < 0.05, **p < 0.01, ***p < 0.001, vs water.

Further, the study of the expression of colonic antibacterial factors revealed that, unlike let-7b, miR-21 significantly increased the expression of the genes *LypD8*, known to bind and inhibit the motility of flagellated bacteria^43^; and Zymogen Granule Protein 16 (*ZG16*), which protein aggregates Gram-positive bacteria^44^ (Figure 5(c)). The mRNA levels of genes encoding for antimicrobial peptides from the defensin family such as cryptidins (*DefCR*)2 and *DefcCR5* were found to be enhanced only in the miR-21 group (Figure 5(c)). No differences were, however, observed in the expression of lysozyme 1 (*Lyz1*), defensin beta (*Defβ)2*, *Defβ10* (Figure 5(c)), nor *Defβ4A* (undetected), which has previously been shown to be targeted by miR-21.^45^

These findings suggest that fecal miRNAs detrimentally affect host-microbiota interaction at the mucosal surface, disturbing intestinal homeostasis. Moreover, the impact of miR-21 on the host seems to be more pronounced than let-7b’s.

### Persistence of let-7b-induced microbiota alterations is associated with delayed miRNA-induced inflammation

**Figure 6.**
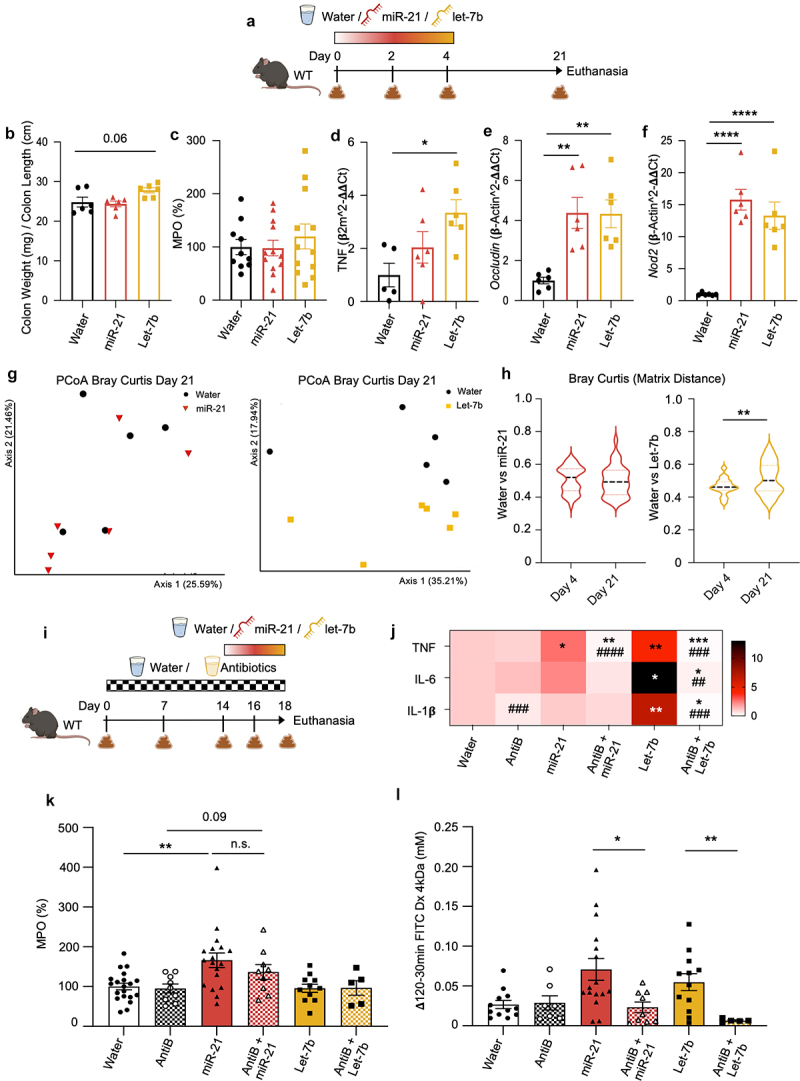
Let-7b-induced inflammation was associated with long-term microbiota alterations and prevented by antibiotics.

To further investigate the resilience of the microbiota and to understand the relationship between these changes and the host inflammation, we maintained mice for 17 days with water after finalizing the 4-day miRNA administration (Figure 6(a)). An almost significant increase in the colon weight/length ratio in let-7b-treated mice was found (Figure 6(b)). Moreover, the above-mentioned acute proinflammatory effects induced by miR-21 were diminished and no longer detected in MPO levels (Figure 6(c)) or in histological analysis (mean of histological scores ± SEM: water 2.33 ± 0.22, miR-21 2.17 ± 0.31, let-7b 2 ± 0.52). In contrast, mRNA expression levels of TNF remained significantly enhanced in the let-7b group (Figure 6d), supporting our findings that let-7b is related to chronic inflammation. The overexpression detected of the key tight junction *occludin* suggests that the intestinal barrier function remained altered (Figure 6e). Furthermore, *Nod2*, known to promote host defense after the detection of muramyl dipeptide (MDP)^46^ and to be upregulated by TNF,^47^ was found significantly increased in all miRNA-treated mice compared to the water-treated group (Figure 6f).

(a) C57Bl/6 wild-type (WT) mice were treated *ad libitum* with water, miR-21 [200 nM], or let-7b [200 nM] for 4 days (*n* = 6 per group). Treatments were then removed and animals were kept only with water for a total of 21 days. (b) Colon weigh/colon length ratio. (c) Colonic amounts of the neutrophil inflammatory marker myeloperoxidase (MPO). (d-f) mRNA expressions levels of TNF (d), tight junction protein occluding (e), and Nod2 (f). (g) PCoA of Bray Curtis of mice microbiota at day 21 previously treated with miR-21 (left) or Let-7b (right). (h) Comparison of the relative Bray Curtis distance matrixes between days 4 and 21 for miR-21 (left) and let-7b treated microbiota (right). (i) WT mice were treated *ad libitum* with a mix of antibiotics (AntiB) for 14 days, before 4-day treatment with water, miR-21, or let-7b (*n* = 9 per group). (j) Heatmap representing colonic mRNA expression levels of proinflammatory cytokines. (k) Colonic myeloperoxidase. (l) Paracellular permeability. Statistical analyses were performed using Mann-Whitney U test or Kruskal-Wallis followed by Dunn’s post hoc tests. Significant differences were recorded as *p < 0.05, **p < 0.01, ***p < 0.001, ****p < 0.0001 when compared to each respective control (mimic *vs*. water, or AntiB + mimic *vs*. AntiB). Comparisons between AntiB-treated groups were done with their related controls without AntiB and significant differences were recorded as #p < 0.05, ##p < 0.01, ###p < 0.001, ####p < 0.0001.

Regarding the intestinal microbiota composition, 16S rRNA gene sequencing followed by the analysis of the beta diversity revealed that the microbiota remained unmodified from day 4 to day 21 in the miR-21 group. Whereas, the microbiota alterations previously found after 4 days of let-7b (as shown in Figure 4) were intensified and kept drifting, becoming significantly different after 17 days without the mimic (Bray Curtis Figure 6g–h, weighted and unweighted Unifrac Supplementary Figures S2d,e).

Overall, these data indicate that the miRNA-induced changes have a prolonged effect on the host inflammatory response. The regression of colitis, when miR-21 was depleted, supports the idea that miR-21’s proinflammatory effects are mostly the consequence of a direct interaction with the host,^48^ rather than through bacterial modifications. These results also suggest that let-7b persistent microbial changes could be the trigger rather than the consequence of the detected inflammation.

### Let-7b-induced inflammation is abrogated in the absence of intestinal microbiota

To further explore whether the inflammatory effects of let-7b and miR-21 in the colon were mediated by direct miRNA-host interactions or as an indirect consequence of the microbiota changes, colitis was studied in intestinal microbiota-depleted mice. WT mice were treated with a mix of antibiotics for 14 days before the 4-day-miRNA administration (Figure 6(i)). Even though miR-21 proinflammatory effects were slightly reduced when the mimic was jointly administered with antibiotics (Figure 6(j), detailed in Supplementary Figure S3a-c), previously shown elevated MPO levels remained increased (Figure 6(k)), suggesting a recruitment of neutrophils independent of the bacterial presence. By contrast, the joint administration of let-7b with antibiotics drastically reduced all the analyzed proinflammatory markers and abolished intestinal inflammation (Figure 6(j), detailed in Supplementary Figure S3a-c). Impaired paracellular permeability was likewise restored, as shown by the passage of FITC-Dx 4 kDa (Figure 6(l)). The antibiotic approach used here had an impact on the basal expression of tight junctions and mucus production, thus further conclusions could not be reached (Supplementary Figure S3d-l).

Even though the microbiota was confirmed to be depleted after 14-days of antibiotic treatment (Supplementary Figure S3m-o), an additional germ-free experiment was performed to evaluate the pro-inflammatory potential of let-7b and miR-21 in complete absence of bacteria (Supplementary Figure S4). As previously performed, wild-type mice were treated for 4 days with either water, let- 7b, or miR-21. Inflammation was studied using macroscopic and molecular parameters, and permeability was assessed *ex vivo* in Ussing chambers. Under germ-free conditions, no inflammation nor an altered permeability was observed with any of the miRNA treatments, supporting that the presence of the microbiota is necessary for the miRNAs to exert their pro-inflammatory potential.

Altogether, these findings suggest that the microbiota plays a crucial role in the observed proinflammatory outcomes of increased luminal miRNAs, especially let-7b.

### Anti-miRNA treatment has an important anti-inflammatory effect through the improvement of intestinal permeability and the prevention of dysbiosis

**Figure 7.**
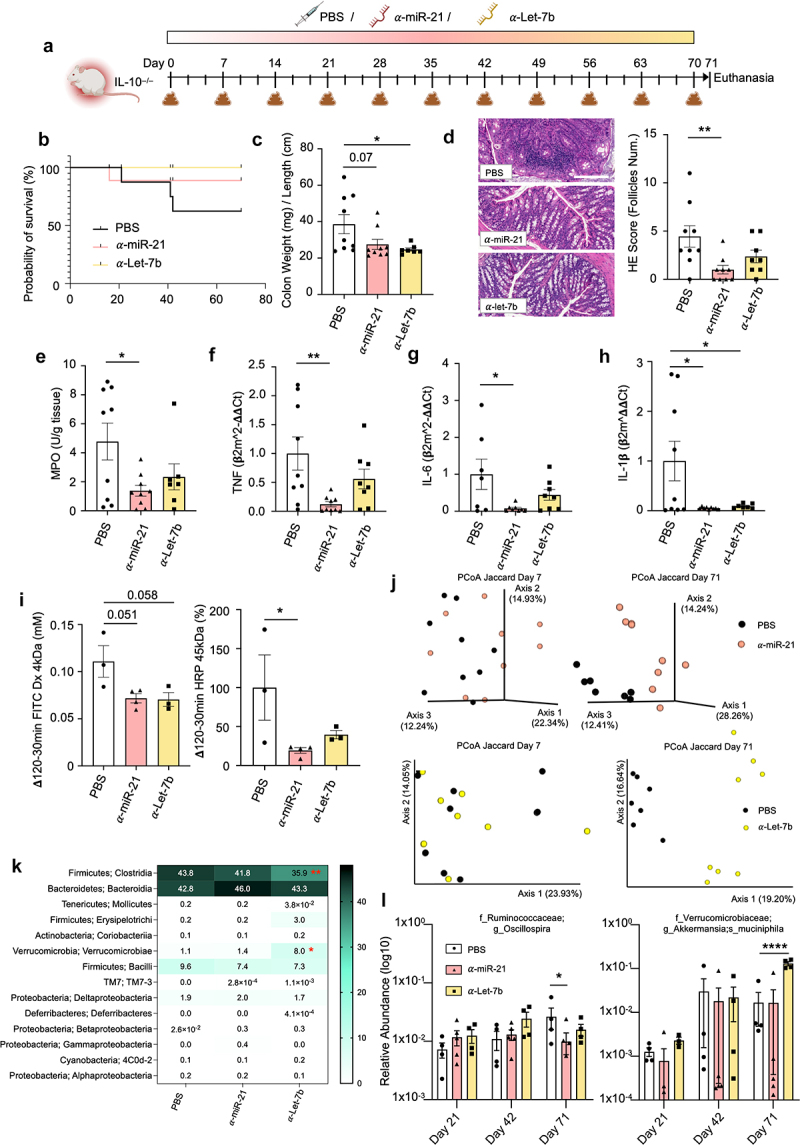
The powerful therapeutic anti-inflammatory potential by inhibiting endogenous miR-21 and let-7b.

Providing the ability of miR-21 and let-7b to modify both the host and the microbiota, thus triggering inflammation and dysbiosis, we next investigated their therapeutic potential by inhibiting endogenous miR-21 and let-7b with anti-miRNAs (Figure 7(a)). Long-term administration of miRNA inhibitors to IL-10^–/–^ mice (from 4 to 15 weeks of age) increased mice’s probability of survival from 62.5% for the PBS control group to 88.8% and 100% for the anti-miR-21 and anti-let-7b treated groups, respectively (Figure 7(b)). Furthermore, administration of both anti-miRNA treatments resulted in a significant reduction of the intrinsic inflammation typically observed in IL-10^–/–^ mice. This was evident from macroscopic measurements, where a notable decrease of the colon weight/colon length ratio was found (Figure 7(c)). Microscopic examination of colonic samples after H&E staining further confirmed the reduced inflammation, which was significant in those mice treated with anti-mir-21 (Figure 7(d)). Similarly, the measurement of the inflammatory neutrophil marker MPO indicated an amelioration in colitis, particularly after anti-miR-21 treatment (Figure 7(e)). Furthermore, the anti-inflammatory effects of both anti-miRNAs were supported by RT-qPCR analysis. The colonic mRNA expression levels of proinflammatory cytokines, including TNF, IL-6, and IL-1β, were drastically downregulated (Figures 7f–h). To further explore the therapeutic potential of miRNA inhibitors, a complementary experiment was performed using another model, this time chemically inducing acute colitis with 1% DSS. Short-term treatment with anti-miR-21 showed comparable anti-inflammatory effects on the acute colitis (Supplementary Figure S6a-g) as those previously observed in IL-10^–/–^ mice. In contrast, anti-let-7b exhibited a less pronounced therapeutic effect. This result aligns with our hypothesis that let-7b’s inflammatory effects may be indirect, by modifying the microbiota. Note that the DSS model induces colitis through direct chemical damage to the epithelial barrier, leading to an acute inflammatory response that is less dependent on the presence of microbiota, unlike the IL10^−/−^ model, which relies heavily on microbiota to trigger chronic inflammation.^49^

(a) IL-10^–/–^ mice (*n* = 9 per group) were orally treated for 10 weeks with miRNA inhibitors. (b) Survival curve during the 10 weeks. (c) Colon weight on colon length ration. (d) The colon of IL-10^–/–^ mice were stained with H&E (bar = 200 μm) and scored. (e) Colonic myeloperoxidase (MPO) amounts. (f-h) Colonic mRNA expression levels of the proinflammatory cytokines TNF (F), IL-6 (g), and IL-1β (H) at day 71. (i) Paracellular and transcellular passage of FITC-Dx and HRP were measured over 120 min in colonic samples mounted in Ussing chambers. (j) Principal coordinates analysis (PCoA) of the Jaccard matrix of anti-miR-21 (top) or anti-let-7b (bottom) treated mice microbiota assessed by 16S rRNA gene sequencing at days 7 (left) and 71 (right). (k) Microbiota samples were compared at the class level after treatment. (l) The two most significantly modified bacterial species were compared per treatment over time. For bar plot graphs, values are given as mean ± SEM. Statistical analyses were performed using Kruskal-Wallis followed by Dunn’s post hoc tests. Significant differences were recorded as *p < 0.05, **p < 0.01, ***p < 0.001, vs PBS. For the heatmap, the color represents the average mean per group.

To assess the extent of the protective effects, we also evaluated the intestinal permeability using FITC Dx 4 kDa and HRP 45 kDa measurements. The results showed a reduction of both paracellular and transcellular permeability (Figure 7(i)), indicating a successful improvement of the intestinal barrier function.

Regarding the microbiota, PCoA analysis revealed anti-miRNAs to significantly alter beta diversity and drove microbiota different from that of untreated mice (Figure 7(j) and Supplementary Figure S5). Moreover, the microbiota composition of mice treated with inhibitors was characterized by a significant decrease of *Firmicutes Clostridia* and an increase of *Verrucomicrobia Verrucomicrobiae* classes after anti-let-7b (Figure 7(k)). We then determined that the most affected bacteria from those classes were *Oscillospira* and *Akkermansia muciniphila* (Figure 7(l)).

Altogether, these results provide strong evidence of the anti-inflammatory potential of the two anti-miRNAs, which exhibited protective properties against inflammation-associated mortality, improved intestinal permeability, and mitigated dysbiosis. Thereby, these *in vivo* data show that the therapeutic inhibition of miR-21 and let-7b might represent effective new approaches in treating colitis. More generally, our findings suggest that miRNAs might be used as effective tools to restore a healthy host-microbiota interaction in chronic intestinal disorders.

## Discussion

**Figure 8.**
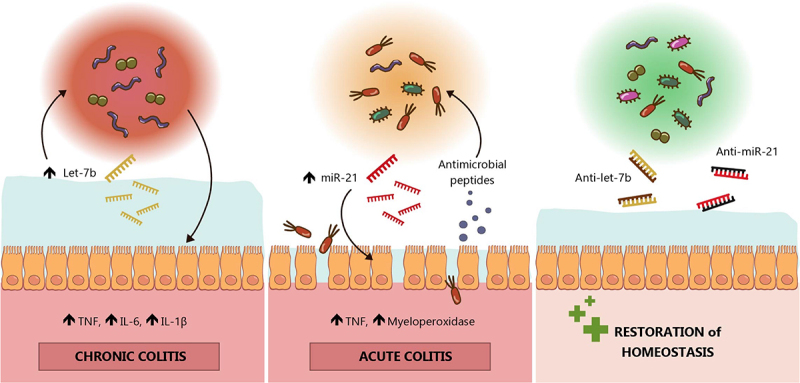
Graphical summary of our key findings.

We and others have previously highlighted the importance played by miRNAs in maintaining intestinal homeostasis.^8,13,29^ However, the relevancy of imbalanced miRNA-microbiota interactions in IBD remains unresolved. Here, we present strong evidence linking both fecal let-7b and miR-21 with colonic inflammation and dysbiosis, both in human and mice. Our findings, summarized on Figure 8, bring novelty not only as they demonstrate that miRNAs can directly impact a complex human microbiota but also as they identified distinct mechanisms of action for let-7b and miR-21, the former primarily reshaping the gut microbiota and the latter impairing the intestinal barrier, both ultimately impacting the inflammatory response. We also show for the first time that inhibiting these endogenous miRNAs notably improves colitis and dysbiosis.

Elevated levels of let-7b in the intestinal lumen strongly reshaped the microbiota and caused persistent changes in the microbiota composition and function. This dysbiosis seemed to trigger low-grade chronic inflammation characterized by more mucus production and upregulated tumor necrosis factor (TNF), interleukin (IL)-6, and IL-1β. Whereas, miR-21 compromised intestinal barrier function and integrity, facilitated bacterial translocation, and thereby induced acute colonic inflammation with increased levels of TNF and myeloperoxidase. Although miRNA-bacterial interactions are possible, miR-21 mild dysbiosis seemed to be primarily associated with the enhanced production of antimicrobial peptides. Additionally, endogenous inhibition of miRNAs during colitis with antisense anti-miR-21 and anti-let-7b had remarkable therapeutic anti-inflammatory effects.

Shreds of evidence relating let-7b with IBD are still scarce, and if any, they are associated with CD.^50–52^ Noteworthy, its mucosal overexpression has been correlated with C-reactive protein (CRP) levels, a biological marker of inflammation.^53^ Here, oral administration of let-7b to WT mice was sufficient to induce low-grade chronic inflammation characterized by the mRNA expression linked with the presence of macrophages. Some studies have reported intestinal macrophages to be the main target of let-7b,^38^ recognized as a potential contributor to their activation, polarization, and inflammatory response.^51^ We also observed that mice treated with let-7b exhibited significant persistent microbiota alterations, alongside aggravated colitis that was drastically suppressed with antibiotic treatment, indicating a reliance on the presence of microbiota. Despite the fact that specific bacteria might persist after the cocktail of antibiotics used in this study,^54^ the utilization of both antibiotic-treated and germ-free models not only addressed this limitation but also strengthened the validation of our findings. We hypothesize that let-7b-induced microbial changes may act as a trigger rather than a consequence of inflammation. This is a new hypothesis and our study provides the first evidence, thus, further research supporting our results is required. Nonetheless, the mucosal overexpression of let-7b has recently been associated with the bacterial invasion of intestinal epithelial cells in CD patients,^50^ and with the activation of NF-κB and the expression of downstream genes involved in the immune responses during *Helicobacter pylori* infection.^55^

MiR-21 is one of the most overexpressed and well-studied miRNAs in both IBD^56^ and colorectal cancer.^57^ A recent meta-analysis has revealed a positive association between miR-21 expression in colon tissue and the development of IBD, suggesting miR-21 as a potential disease marker.^56,58^ In the context of murine colitis, we^19^ and others^59^ have shown increased miR-21 levels in IL-10^–/–^ mice. Complementary, other studies have reported that the deletion of miR-21 results in the reduction of susceptibility to induced experimental colitis.^60,61^ In the present study, the luminal increase of miR-21 in WT mice was enough to enhance bacterial penetration and impairment of the intestinal barrier function, with the main alteration of claudins. Supporting our results, the overexpression of miR-21 has previously been described to regulate intestinal epithelial tight junctions through the degradation of RhoB mRNA^62^ and the upregulation of ADP ribosylation factor 4 (ARF4)^63^ in the PTEN/PI3K/Akt signaling pathway.^48^ Here, miR-21 also induced an acute low-grade inflammation characterized by increased MPO and the enhancement of antimicrobial peptides, possibly contributing to the mild changes in the microbiota composition and function. The lack of obvious changes on MPO in miR-21 treated mice aligns with the other findings presented in this study and further indicates that the pro-inflammatory effect of let-7b is more dependent on the presence of the microbiota than miR-21’s. Exploring how miRNAs might be influencing MPO levels, even in the presence of antibiotics, could lead to a more comprehensive discussion and highlight the different pro-inflammatory molecular mechanisms. Johnston *et al*. previously reported that the protective effects shown in miR-21^–/–^ mice are, at least in part, dependent on the presence of the characteristic microbiota.^64^ However, the authors did not account for the indirect effects on the microbiota by miR-21-induction of defensins nor with direct miRNA-microbiota interactions. In line with our *in vivo* results, and as further discussed below, we show novel bacterial changes in complex microbiota when incubating it with miR-21.

Recent studies have established the presence of miRNAs in feces and have suggested that these molecules can enter bacteria and modulate gene expression, affecting bacterial growth and metabolites.^13,45,65^ Using a unique anaerobic *in vitro* culture model of human-derived microbiota,^27,36,66^ we found specific miRNA-bacterial interactions and modulatory impact on the composition of complex gut microbiota, influencing its diversity, composition, and proinflammatory potential. Consistent with our findings, a previous study showed that the RNA cargo of milk exosomes alters the gut microbiota composition, without, however, specifying which miRNAs were carried.^67^ Our correlation analyses in human samples shed light on the association between luminal miRNAs and various bacterial species. Notably, several genus from the *Lachnospiraceae* family were found here decreased after incubation with miRNAs and negatively correlated with miR-21. A loss in taxa belonging to the family of *Lachnospiraceae*, especially of the genus *Blautia* and *Ruminococcus*, has been repeatedly implicated in IBD.^68,69^ We acknowledge the limitations of the MBRA system, particularly regarding the potential loss of certain bacterial species, such as mucus-dependent bacteria. Due to practical limitations related to the quantity, preservation, and cultivation of human samples, as well as space constraints within the system, we did not use miR control. Instead, we used PBS-treated group as a control considering that two different miRNAs would allow us to consider them as controls for each other and we tested samples from two different donors (donor 1 and 2) in parallel to address interindividual variations, providing a robust control for assessing the miRNA effect. Our *in vivo* results showed a mucus production shift after miRNA treatment, accompanied by an increased encroachment of bacteria. To address this, future studies could introduce mucin beads in the system,^70^ thereby promoting the persistence and growth of mucus-degrading bacteria and enabling the study of bacteria such as *Akkermansia muciniphila*, which was found remarkably increased after inhibition of let-7b *in vivo*.

Having revealed the pathogenic potential of miR-21 and let-7b, we then postulated that their inhibition with antisense oligonucleotides (ASO) may have beneficial effects, as previously shown,^71^ correcting abnormal functions.^72,73^ Here, the long-term administration of either anti-miR-21 or anti-let-7b to IL-10 ^–/–^ mice provided a protective effect with increased survival. Moreover, the inhibition of endogenous miRNAs helped maintain intestinal barrier integrity and reduced the passage of potentially harmful substances. Regarding the intestinal microbiota, anti-miRNA treatments caused a shift in the overall composition of the microbiota, being more drastic for those mice treated with anti-let-7b, preventing the development of dysbiosis. To our knowledge, our study provides the first evidence of the *in vivo* therapeutic approach inhibiting upregulated let-7b and miR-21 in the context of colitis. The closest research, performed by Ding *et al*., reported the use of ASO against miR-21 in a xenograft model of colorectal carcinoma that effectively inhibited tumor growth and metastasis.^74^

In conclusion, we unrevealed new and specific inter-kingdom communications for two colitis-associated miRNAs, let-7b and miR-21. While miR-21 had intense detrimental effects on the host and minor dysbiosis; let-7b inflammatory response was strongly associated with microbiota alterations. This discovery opens up a promising avenue for future research, focusing on elucidating the mechanisms underlying the interactions between luminal miRNA and bacteria. Additionally, endogenous inhibition of miRNAs during colitis with anti-miR-21 and anti-let-7b had remarkable therapeutic anti-inflammatory effects. Overall, this work advances our understanding of fecal miRNA dynamics and their potential implications in intestinal health, offering insights into their multifaceted roles in dysbiosis-related conditions and intestinal inflammation, particularly relevant in IBD.

## Supplementary Material

Supplemental Material

## Data Availability

Unprocessed sequencing data are deposited in the European Nucleotide Archive under accession number PRJEB66130.

## References

[1] Xavier RJ, Podolsky DK. Unravelling the pathogenesis of inflammatory bowel disease. Nature. 2007;448(7152):427–25. doi:10.1038/nature06005. 17653185

[2] Qin J, Li R, Raes J, Arumugam M, Burgdorf KS, Manichanh C, Nielsen T, Pons N, Levenez F, Yamada T, et al. A human gut microbial gene catalogue established by metagenomic sequencing. Nature. 2010;464(7285):59–65. doi:10.1038/nature08821. 20203603 PMC3779803

[3] Kang SS, Bloom SM, Norian LA, Geske MJ, Flavell RA, Stappenbeck TS, Allen PM. An antibiotic-responsive mouse model of fulminant ulcerative colitis. PLOS Med. 2008;5(3):e41. doi:10.1371/journal.pmed.0050041. 18318596 PMC2270287

[4] Nagao-Kitamoto H, Shreiner AB, Gillilland MG, Kitamoto S, Ishii C, Hirayama A, Kuffa P, El-Zaatari M, Grasberger H, Seekatz AM, et al. Functional characterization of inflammatory bowel disease–associated gut dysbiosis in gnotobiotic mice. Cell Mol Gastroenterol Hepatol. 2016;2(4):468–481. doi:10.1016/j.jcmgh.2016.02.003. 27795980 PMC5042563

[5] Kühn R, Löhler J, Rennick D, Rajewsky K, Müller W. Interleukin-10-deficient mice develop chronic enterocolitis. Cell. 1993;75(2):263–274. doi:10.1016/0092-8674(93)80068-p. 8402911

[6] Yau TO, Tang CM, Harriss EK, Dickins B, Polytarchou C. Faecal microRNAs as a non-invasive tool in the diagnosis of colonic adenomas and colorectal cancer: a meta-analysis. Sci Rep. 2019;9(1):9491. doi:10.1038/s41598-019-45570-9. 31263200 PMC6603164

[7] Ji Y, Li X, Zhu Y, Li N, Zhang N, Niu M. Faecal microRNA as a biomarker of the activity and prognosis of inflammatory bowel diseases. Biochem Biophys Res Commun. 2018;503(4):2443–2450. doi:10.1016/j.bbrc.2018.06.174. 29969632

[8] Shen Q, Huang Z, Ma L, Yao J, Luo T, Zhao Y, Xiao Y, Jin Y. Extracellular vesicle miRNAs promote the intestinal microenvironment by interacting with microbes in colitis. Gut Microbes. 2022;14(1):2128604. doi:10.1080/19490976.2022.2128604. 36176029 PMC9542864

[9] Viennois E, Zhao Y, Han MK, Xiao B, Zhang M, Prasad M, Wang L, Merlin D. Serum miRNA signature diagnoses and discriminates murine colitis subtypes and predicts ulcerative colitis in humans. Sci Rep. 2017;7(1):2520. doi:10.1038/s41598-017-02782-1. 28566745 PMC5451415

[10] Fasseu M, Tréton X, Guichard C, Pedruzzi E, Cazals-Hatem D, Richard C, Aparicio T, Daniel F, Soulé J-C, Moreau R, et al. Identification of restricted subsets of mature microRNA abnormally expressed in inactive colonic mucosa of patients with inflammatory bowel disease. PLOS ONE. 2010;5(10):e13160. doi:10.1371/journal.pone.0013160. 20957151 PMC2950152

[11] Iborra M, Bernuzzi F, Correale C, Vetrano S, Fiorino G, Beltrán B, Marabita F, Locati M, Spinelli A, Nos P, et al. Identification of serum and tissue micro-rna expression profiles in different stages of inflammatory bowel disease. Clin Exp Immunol. 2013;173(2):250–258. doi:10.1111/cei.12104. 23607522 PMC3722925

[12] Schönauen K, Le N, von Arnim U, Schulz C, Malfertheiner P, Link A. Circulating and fecal microRNAs as biomarkers for Inflamm bowel Dis. Inflamm Bowel Dis. 2018;24(7):1547–1557. doi:10.1093/ibd/izy046. 29668922

[13] Liu S, da Cunha A, Rezende R, Cialic R, Wei Z, Bry L, Comstock L, Gandhi R, Weiner H. The Host shapes the gut microbiota via fecal MicroRNA. Cell Host Microbe. 2016;19(1):32–43. doi:10.1016/j.chom.2015.12.005. 26764595 PMC4847146

[14] Dalmasso G, Nguyen HTT, Yan Y, Laroui H, Charania MA, Ayyadurai S, Sitaraman SV, Merlin D. Microbiota modulate host gene expression via microRNAs. PLOS ONE. 2011;6(4):e19293. doi:10.1371/journal.pone.0019293. 21559394 PMC3084815

[15] Maudet C, Mano M, Eulalio A. MicroRNAs in the interaction between host and bacterial pathogens. FEBS Lett. 2014;588(22):4140–4147. doi:10.1016/j.febslet.2014.08.002. 25128459

[16] Williams MR, Stedtfeld RD, Tiedje JM, Hashsham SA. MicroRNAs-based inter-domain communication between the host and members of the gut microbiome. Front Microbiol. 2017;8:1896. doi:10.3389/fmicb.2017.01896. 29021788 PMC5624305

[17] Tomkovich S, Gharaibeh RZ, Dejea CM, Pope JL, Jiang J, Winglee K, Gauthier J, Newsome RC, Yang Y, Fodor AA, et al. Human colon mucosal biofilms and murine Host communicate via altered mRNA and microRNA expression during cancer. mSystems. 2020;5(1). doi:10.1128/mSystems.00451-19. PMC6967385 31937674

[18] Ma L, Hou C, Yang H, Chen Q, Lyu W, Wang Z, Wang J, Xiao Y. Multi-omics analysis reveals the interaction of gut microbiome and host microRNAs in ulcerative colitis. Ann Med. 2023;55(2):2261477. doi:10.1080/07853890.2023.2261477. 37774039 PMC10543339

[19] Casado-Bedmar M, Roy M, Viennois E. The effect of sex-specific differences on IL-10−/− mouse colitis phenotype and microbiota. Int J Mol Sci. 2023;24(12):10364. doi:10.3390/ijms241210364. 37373511 PMC10299321

[20] Snoussi C, Ducroc R, Hamdaoui MH, Dhaouadi K, Abaidi H, Cluzeaud F, Nazaret C, Le Gall M, Bado A. Green tea decoction improves glucose tolerance and reduces weight gain of rats fed normal and high-fat diet. J Nutr Biochem. 2014;25(5):557–564. doi:10.1016/j.jnutbio.2014.01.006. 24656388

[21] Keita AV, Gullberg E, Ericson A-C, Salim SY, Wallon C, Kald A, Artursson P, Söderholm JD. Characterization of antigen and bacterial transport in the follicle-associated epithelium of human ileum. Lab Invest. 2006;86(5):504–516. doi:10.1038/labinvest.3700397. 16482102

[22] Ganda Mall JP, Casado-Bedmar M, Winberg ME, Brummer RJ, Schoultz I, Keita ÅV. A β-glucan-based dietary fiber reduces mast cell-induced hyperpermeability in ileum from patients with Crohn’s disease and control subjects. Inflamm Bowel Dis. 2017;24(1):166–178. doi:10.1093/ibd/izx002. 29272475 PMC6166688

[23] Singh V, Yeoh BS, Chassaing B, Xiao X, Saha P, Aguilera Olvera R, Lapek JD, Zhang L, Wang W-B, Hao S, et al. Dysregulated microbial fermentation of soluble fiber induces cholestatic liver cancer. Cell. 2018;175(3):679–694.e622. doi:10.1016/j.cell.2018.09.004. 30340040 PMC6232850

[24] Katakura K, Lee J, Rachmilewitz D, Li G, Eckmann L, Raz E. Toll-like receptor 9–induced type I IFN protects mice from experimental colitis. J Clin Invest. 2005;115(3):695–702. doi:10.1172/jci22996. 15765149 PMC1051992

[25] Viennois E, Xiao B, Ayyadurai S, Wang L, Wang PG, Zhang Q, Chen Y, Merlin D. Micheliolide, a new sesquiterpene lactone that inhibits intestinal inflammation and colitis-associated cancer. Lab Invest. 2014;94(9):950–965. doi:10.1038/labinvest.2014.89. 25068660

[26] Naimi S, Viennois E, Gewirtz AT, Chassaing B. Direct impact of commonly used dietary emulsifiers on human gut microbiota. Microbiome. 2021;9(1):66. doi:10.1186/s40168-020-00996-6. 33752754 PMC7986288

[27] Auchtung JM, Robinson CD, Britton RA. Cultivation of stable, reproducible microbial communities from different fecal donors using minibioreactor arrays (MBRAs). Microbiome. 2015;3(42). doi:10.1186/s40168-015-0106-5. PMC4588258 26419531

[28] Chassaing B, Ley RE, Gewirtz AT. Intestinal epithelial cell toll-like receptor 5 regulates the intestinal microbiota to prevent low-grade inflammation and metabolic syndrome in mice. Gastroenterology. 2014;147(6):1363–1377.e1317. doi:10.1053/j.gastro.2014.08.033. 25172014 PMC4253564

[29] Viennois E, Chassaing B, Tahsin A, Pujada A, Wang L, Gewirtz AT, Merlin D. Host-derived fecal microRNAs can indicate gut microbiota healthiness and ability to induce inflammation. Theranostics. 2019;9(15):4542–4557. doi:10.7150/thno.35282. 31285778 PMC6599659

[30] Johansson ME, Hansson GC. Preservation of mucus in histological sections, immunostaining of mucins in fixed tissue, and localization of bacteria with FISH. Methods Mol Biol. 2012;842:229–235. doi:10.1007/978-1-61779-513-8_13. 22259139

[31] McDonald D, Price MN, Goodrich J, Nawrocki EP, DeSantis TZ, Probst A, Andersen GL, Knight R, Hugenholtz P. An improved Greengenes taxonomy with explicit ranks for ecological and evolutionary analyses of bacteria and archaea. The ISME J. 2012;6(3):610–618. doi:10.1038/ismej.2011.139. 22134646 PMC3280142

[32] Mallick H, Rahnavard A, McIver LJ, Ma S, Zhang Y, Nguyen LH, Tickle TL, Weingart G, Ren B, Schwager EH, et al. Multivariable association discovery in population-scale meta-omics studies. PLOS Comput Biol. 2021;17(11):e1009442. doi:10.1371/journal.pcbi.1009442. 34784344 PMC8714082

[33] Lopetuso LR, Petito V, Graziani C, Schiavoni E, Paroni Sterbini F, Poscia A, Gaetani E, Franceschi F, Cammarota G, Sanguinetti M, et al. Gut microbiota in health, diverticular disease, irritable bowel syndrome, and inflammatory bowel diseases: time for microbial Marker of gastrointestinal disorders. Dig Dis. 2018;36(1):56–65. doi:10.1159/000477205. 28683448

[34] Carey MA, Medlock GL, Alam M, Kabir M, Uddin MJ, Nayak U, Papin J, Faruque ASG, Haque R, Petri WA, et al. Megasphaera in the stool microbiota is negatively associated with diarrheal cryptosporidiosis. Clin Infect Dis. 2021;73(6):e1242–e1251. doi:10.1093/cid/ciab207. 33684930 PMC8442784

[35] Sokol H, Pigneur B, Watterlot L, Lakhdari O, Bermúdez-Humarán LG, Gratadoux J-J, Blugeon S, Bridonneau C, Furet J-P, Corthier G, et al. Faecalibacterium prausnitzii is an anti-inflammatory commensal bacterium identified by gut microbiota analysis of Crohn disease patients. Proc Natl Acad Sci USA. 2008;105(43):16731–16736. doi:10.1073/pnas.0804812105. 18936492 PMC2575488

[36] Auchtung JM, Robinson CD, Farrell K, Britton RA. MiniBioReactor arrays (MBRAs) as a tool for studying C. difficile physiology in the presence of a complex community. Methods Mol Biol. 2016;1476:235–258. doi:10.1007/978-1-4939-6361-4_18. 27507346

[37] Alam MT, Amos GCA, Murphy ARJ, Murch S, Wellington EMH, Arasaradnam RP. Microbial imbalance in inflammatory bowel disease patients at different taxonomic levels. Gut Pathog. 2020;12(1). doi:10.1186/s13099-019-0341-6. PMC6942256 31911822

[38] Xu Y, Qian W, Huang L, Wen W, Li Y, Guo F, Zhu Z, Li Z, Gong J, Yu Z, et al. Crohn’s disease-associated AIEC inhibiting intestinal epithelial cell-derived exosomal let-7b expression regulates macrophage polarization to exacerbate intestinal fibrosis. Gut Microbes. 2023;15(1):2193115. doi:10.1080/19490976.2023.2193115. 36945126 PMC10038049

[39] Jones GR, Bain CC, Fenton TM, Kelly A, Brown SL, Ivens AC, Travis MA, Cook PC, MacDonald AS. Dynamics of colon monocyte and macrophage activation during colitis. Front Immunol. 2018;9:2764. doi:10.3389/fimmu.2018.02764. 30542349 PMC6277765

[40] Lau D, Mollnau H, Eiserich JP, Freeman BA, Daiber A, Gehling UM, Brümmer J, Rudolph V, Münzel T, Heitzer T, et al. Myeloperoxidase mediates neutrophil activation by association with CD11b/CD18 integrins. Proc Natl Acad Sci. 2005;102(2):431–436. doi:10.1073/pnas.0405193102. 15625114 PMC544285

[41] Weber CR, Nalle SC, Tretiakova M, Rubin DT, Turner JR. Claudin-1 and claudin-2 expression is elevated in inflammatory bowel disease and may contribute to early neoplastic transformation. Lab Invest. 2008;88(10):1110–1120. doi:10.1038/labinvest.2008.78. 18711353 PMC2586671

[42] Viennois E, Pujada A, Sung J, Yang C, Gewirtz AT, Chassaing B, Merlin D. Impact of PepT1 deletion on microbiota composition and colitis requires multiple generations. NPJ Biofilms Microbiomes. 2020;6(1):27. doi:10.1038/s41522-020-0137-y. 32694535 PMC7374158

[43] Okumura R, Kurakawa T, Nakano T, Kayama H, Kinoshita M, Motooka D, Gotoh K, Kimura T, Kamiyama N, Kusu T, et al. Lypd8 promotes the segregation of flagellated microbiota and colonic epithelia. Nature. 2016;532(7597):117–121. doi:10.1038/nature17406. 27027293

[44] Bergström JH, Birchenough GMH, Katona G, Schroeder BO, Schütte A, Ermund A, Johansson MEV, Hansson GC. Gram-positive bacteria are held at a distance in the colon mucus by the lectin-like protein ZG16. Proc Natl Acad Sci USA. 2016;113(48):13833–13838. doi:10.1073/pnas.1611400113. 27849619 PMC5137749

[45] Liu PT, Wheelwright M, Teles R, Komisopoulou E, Edfeldt K, Ferguson B, Mehta MD, Vazirnia A, Rea TH, Sarno EN, et al. MicroRNA-21 targets the vitamin D–dependent antimicrobial pathway in leprosy. Nat Med. 2012;18(2):267–273. doi:10.1038/nm.2584. 22286305 PMC3274599

[46] Girardin SE, Boneca IG, Viala J, Chamaillard M, Labigne A, Thomas G, Philpott DJ, Sansonetti PJ. Nod2 is a general sensor of peptidoglycan through Muramyl Dipeptide (MDP) Detection*. J Biol Chem. 2003;278(11):8869–8872. doi:10.1074/jbc.C200651200. 12527755

[47] Hisamatsu T, Suzuki, M., Reinecker, H.C., Nadeau, W.J., McCormick, B.A., & Podolsky, D.K. CARD15/NOD2 functions as an antibacterial factor in human intestinal epithelial cells. Gastroenterology. 2003;124(4):993–1000. doi:10.1053/gast.2003.50153. 12671896

[48] Zhang L, Shen J, Cheng J, Fan X. MicroRNA-21 regulates intestinal epithelial tight junction permeability. Cell Biochem Funct. 2015;33(4):235–240. doi:10.1002/cbf.3109. 25997617

[49] Wirtz S, Neurath MF. Mouse models of inflammatory bowel disease. Adv Drug Delivery Rev. 2007;59(11):1073–1083. doi:10.1016/j.addr.2007.07.003. 17825455

[50] Fernández-Ponce C, Navarro Quiroz R, Díaz Perez A, Aroca Martinez G, Cadena Bonfanti A, Acosta Hoyos A, Gómez Escorcia L, Hernández Agudelo S, Orozco Sánchez C, Villarreal Camacho J, et al. MicroRNAs overexpressed in Crohn’s disease and their interactions with mechanisms of epigenetic regulation explain novel aspects of Crohn’s disease pathogenesis. Clin Epigenet. 2021;13(1):39. doi:10.1186/s13148-021-01022-8. PMC7890887 33602320

[51] Gong L, Xiao J, Yi J, Xiao J, Lu F, Liu X. Immunomodulatory effect of serum exosomes from crohn disease on macrophages via let-7b-5p/TLR4 signaling. Inflamm Bowel Dis. 2021;28(1):96–108. doi:10.1093/ibd/izab132. 34106260

[52] Zahm AM, Thayu M, Hand NJ, Horner A, Leonard MB, Friedman JR. Circulating microRNA is a biomarker of pediatric Crohn disease. J Pediatr Gastroenterol Nutr. 2011;53(1):26–33. doi:10.1097/MPG.0b013e31822200cc. 21546856 PMC3807879

[53] Guo Z, Gong J, Li Y, Gu L, Cao L, Wang Z, Zhu W, Li J. Mucosal MicroRNAs expression profiles before and after exclusive enteral nutrition therapy in adult patients with Crohn’s disease. Nutrients. 2016;8(8):519. doi:10.3390/nu8080519. 27556489 PMC4997431

[54] Tan J, Gong J, Liu F, Li B, Li Z, You J, He J, Wu S. Evaluation of an antibiotic cocktail for fecal microbiota transplantation in mouse. Front Nutr. 2022;9:918098. doi:10.3389/fnut.2022.918098. 35719145 PMC9204140

[55] Teng GG, Wang W-H, Dai Y, Wang S-J, Chu Y-X, Li J. Let-7b is involved in the inflammation and immune responses associated with Helicobacter pylori infection by targeting Toll-like receptor 4. PLOS ONE. 2013;8(2):e56709. doi:10.1371/journal.pone.0056709. 23437218 PMC3577724

[56] Yan H, Zhang X, Xu Y. Aberrant expression of miR-21 in patients with inflammatory bowel disease: a protocol for systematic review and meta analysis. Med (Baltim). 2020;99(17):e19693. doi:10.1097/md.0000000000019693. PMC7220677 32332611

[57] You C, Jin L, Xu Q, Shen B, Jiao X, Huang X. Expression of miR‑21 and miR‑138 in colon cancer and its effect on cell proliferation and prognosis. Oncol Lett. 2019;17:2271–2277. doi:10.3892/ol.2018.9864. 30675293 PMC6341732

[58] Zhou R, Qiu P, Wang H, Yang H, Yang X, Ye M, Wang F, Zhao Q. Identification of microRNA-16-5p and microRNA-21-5p in feces as potential noninvasive biomarkers for inflammatory bowel disease. Aging (Albany NY). 2021;13(3):4634–4646. doi:10.18632/aging.202428. 33535181 PMC7906140

[59] Schaefer JS, Montufar-Solis D, Vigneswaran N, Klein JR. Selective upregulation of microRNA expression in peripheral blood leukocytes in IL-10−/− mice precedes expression in the colon. J Immunol. 2011;187(11):5834–5841. doi:10.4049/jimmunol.1100922. 22043014 PMC3221883

[60] Shi C, Liang Y, Yang J, Xia Y, Chen H, Han H, Yang Y, Wu W, Gao R, Qin H, et al. MicroRNA-21 knockout improve the survival rate in DSS induced fatal colitis through protecting against inflammation and tissue injury. PLOS ONE. 2013;8(6):e66814. doi:10.1371/journal.pone.0066814. 23826144 PMC3691313

[61] Wu F, Dong F, Arendovich N, Zhang J, Huang Y, Kwon JH. Divergent influence of microRNA-21 deletion on murine colitis phenotypes. Inflamm Bowel Dis. 2014;20(11):1972–1985. doi:10.1097/mib.0000000000000201. 25222661

[62] Yang Y, Ma Y, Shi C, Chen H, Zhang H, Chen N, Zhang P, Wang F, Yang J, Yang J, et al. Overexpression of miR-21 in patients with ulcerative colitis impairs intestinal epithelial barrier function through targeting the rho GTPase RhoB. Biochem Biophys Res Commun. 2013;434(4):746–752. doi:10.1016/j.bbrc.2013.03.122. 23583411

[63] Nakata K, Sugi Y, Narabayashi H, Kobayakawa T, Nakanishi Y, Tsuda M, Hosono A, Kaminogawa S, Hanazawa S, Takahashi K, et al. Commensal microbiota-induced microRNA modulates intestinal epithelial permeability through the small GTPase ARF4. J Biol Chem. 2017;292(37):15426–15433. doi:10.1074/jbc.M117.788596. 28760826 PMC5602400

[64] Johnston DGW, Williams MA, Thaiss CA, Cabrera-Rubio R, Raverdeau M, McEntee C, Cotter PD, Elinav E, O’Neill LAJ, Corr SC, et al. Loss of MicroRNA-21 influences the gut microbiota, causing reduced susceptibility in a murine Model of colitis. J Crohns Colitis. 2018;12(7):835–848. doi:10.1093/ecco-jcc/jjy038. 29608690

[65] Teng Y, Ren YI, Sayed M, Hu X, Lei C, Kumar A, Hutchins E, Mu J, Deng Z, Luo C, et al. Plant-derived Exosomal MicroRNAs shape the gut microbiota. Cell Host & Microbe. 2018;24:637–652.e638. doi:10.1016/j.chom.2018.10.001. 30449315 PMC6746408

[66] Hobson CA, Vigue L, Naimi S, Chassaing B, Magnan M, Bonacorsi S, Gachet B, El Meouche I, Birgy A, Tenaillon O, et al. MiniBioReactor array (MBRA) in vitro gut model: a reliable system to study microbiota-dependent response to antibiotic treatment. JAC-Antimicrob Resist. 2022;4(4). doi:10.1093/jacamr/dlac077. PMC9252984 35795241

[67] Zhou F, Paz HA, Sadri M, Cui J, Kachman SD, Fernando SC, Zempleni J. Dietary bovine milk exosomes elicit changes in bacterial communities in C57BL/6 mice. Am J Physiol Gastrointest Liver Physiol. 2019;317(5):G618–g624. doi:10.1152/ajpgi.00160.2019. 31509432 PMC6879888

[68] Gevers D, Kugathasan S, Denson L, Vázquez-Baeza Y, Van Treuren W, Ren B, Schwager E, Knights D, Song S, Yassour M, et al. The treatment-naive microbiome in new-onset Crohn’s disease. Cell Host Microbe. 2014;15(3):382–392. doi:10.1016/j.chom.2014.02.005. 24629344 PMC4059512

[69] Sasaki K, Inoue J, Sasaki D, Hoshi N, Shirai T, Fukuda I, Azuma T, Kondo A, Osawa R. Construction of a Model culture system of human colonic microbiota to detect decreased Lachnospiraceae abundance and butyrogenesis in the feces of ulcerative colitis patients. Biotechnol J. 2019;14(5):e1800555. doi:10.1002/biot.201800555. 30791234

[70] Sauvaitre T, Van Landuyt J, Durif C, Roussel C, Sivignon A, Chalancon S, Uriot O, Van Herreweghen F, Van de Wiele T, Etienne-Mesmin L, et al. Role of mucus-bacteria interactions in enterotoxigenic Escherichia coli (ETEC) H10407 virulence and interplay with human microbiome. NPJ Biofilms Microbiomes. 2022;8(1). doi:10.1038/s41522-022-00344-6. PMC9584927 36266277

[71] Lima JF, Cerqueira L, Figueiredo C, Oliveira C, Azevedo NF. Anti-miRNA oligonucleotides: a comprehensive guide for design. RNA Biol. 2018;15(3):338–352. doi:10.1080/15476286.2018.1445959. 29570036 PMC5927725

[72] Casado-Bedmar M, Viennois E. MicroRNA and gut microbiota: tiny but mighty—novel insights into their cross-talk in inflammatory bowel disease pathogenesis and therapeutics. J Crohns Colitis. 2022;16(6):992–1005. doi:10.1093/ecco-jcc/jjab223. 34918052 PMC9282881

[73] Zhu F, Li H, Liu Y, Tan C, Liu X, Fan H, Wu H, Dong Y, Yu T, Chu S, et al. miR-155 antagomir protect against dss-induced colitis in mice through regulating Th17/Treg cell balance by Jarid2/Wnt/β-catenin. Biomed Pharmacother. 2020;126:109909. doi:10.1016/j.biopha.2020.109909. 32135463

[74] Ding T, Cui P, Zhou Y, Chen C, Zhao J, Wang H, Guo M, He Z, Xu L. Antisense Oligonucleotides against miR-21 inhibit the growth and metastasis of colorectal carcinoma via the DUSP8 pathway. Mol Ther Nucleic Acids. 2018;13:244–255. doi:10.1016/j.omtn.2018.09.004. 30317164 PMC6187053

