# Supplementary material for: Fecal let-7b and miR-21 directly modulate the intestinal microbiota, driving chronic inflammation
Source: Gut Microbes. 2024 Sep 3;16(1):2394249. doi: 10.1080/19490976.2024.2394249 (PMC11376420; doi:10.1080/19490976.2024.2394249)

**Supplementary Figure Legends**

**Supplementary Figure S1. Human microbiota and schematic representation of MBRA experimental design. (A)** Taxonomic representation with Lefse and **(B)** alpha diversity analysis comparing the microbiota from healthy controls and inflammatory bowel diseases patients. **(C)** Fecal samples from 2 healthy human donors were inoculated and treated (in triplicate) with miRNAs after 120 h of stabilization in an MiniBioReactor Arrays (MBRA) system. **(D)** Relative abundance of *Clostridia Clostridiales Lachnospiraceae Blautia* and *Ruminococcus* at class, family, and genus level of human samples incubated with either let-7b or miR-21 for 60 h.

**Supplementary Figure S2. Study of the viability and specificity of miRNAs and effect on the microbiota. (A)** The amounts of miR-21 and let-7b were measured after being diluted in water at a final concentration of 200nM and kept at room temperature for 4 days in the drinking bottles. **(B-C)** A pilot study was performed to test for off-target effects and specificity. C57Bl/6 wild-type (WT) mice were treated for 4 days in the drinking water *ad libitum* with either water or control miRNA (Qiagen). Colonic inflammatory effects were measured on day 4. **(B)** Colonic myeloperoxidase (MPO) amounts were measured and normalized to the water group. **(C)** Relative amounts of mRNA expression of the pro-inflammatory cytokines TNF, IL-6 and IL-1β were studied by RT-qPCR. Data are represented as mean ± SEM. Statistical analyses were performed using the Mann-Whitney U test. **(D-E)** C57Bl/6 wild-type (WT) mice were treated ad libitum with water, miR-21 [200nM], or let-7b [200nM] for 4 days (n=6 per group). Treatments were then removed and animals were kept only with water for a total of 21 days (see Figure 6I). PCoA of the **(D)** weighted and **(E)** unweighted Unifrac distance matrix of miR-21 (top) and let-7b (bottom) treated mice microbiota at day 21.

**Supplementary Figure S3. Let-7b-induced inflammation was prevented by antibiotics.** C57Bl/6 wild-type (WT) mice were treated *ad libitum* with a mix of antibiotics (AntiB) for 14 days, before the 4-day treatment with either water, miR-21 [200nM], or let-7b [200nM] for 4 days. The mRNA expressions levels were measured by RT-qPCR in colonic samples at day 18. **(A-C)** Pro-inflammatory cytokines TNF, IL-6, and IL-1β. **(D-I)** Tight junction proteins. **(J-L)** Mucus proteins. **(M, N)** Lipopolysaccharide (LPS) and flagellin measurements using TLR4-HEK cells. **(O)** Bacterial DNA load. Values are given as mean ± SEM. Statistical analyses were performed using Mann-Whitney U test or Kruskal-Wallis followed by Dunn’s post hoc tests. Significant differences were recorded as *p<0.05, **p<0.01, ***p<0.001 when compared to each respective control (mimic *vs.* water, or AntiB + mimic *vs.* AntiB). Comparisons between AntiB-treated groups were done with their related controls without AntiB and significant differences were recorded as #p<0.05, ##p<0.01, ###p<0.001, ####p<0.0001.

**Supplementary Figure S4. miR-induced inflammation was abolished under germ-free conditions.** C57Bl/6 wild-type germ-free mice were treated *ad libitum* with either water (n=4), miR-21 [200nM] (n=4), or let-7b [200nM] (n=4) for 4 days. **(A)** Colon weight/colon length ratio. **(B-D)** Relative amounts of mRNA expression of the pro-inflammatory cytokines **(B)** TNF, **(C)** IL-6 and **(D)** IL-1β were studied by RT-qPCR. **(E)** Colonic myeloperoxidase (MPO) amounts were measured and normalized to the water group. **(F)** Paracellular passage of FITC-Dx was measured over 120 min in colonic samples mounted in Ussing chambers. Data are represented as mean ± SEM. Statistical analyses were performed using the Mann-Whitney U test.

**Supplementary Figure S5.** The Jaccard distance separating mice samples after anti-miR-21 (left) and anti-let-7b (right) treatment at day 71. Data are represented in box plots as median ± quartiles. Statistical analyses were performed using a Kruskal-Wallis followed by a Dunn’s post hoc test *vs.* PBS-PBS distance.

**Supplementary Figure S6. Therapeutic potential during acute colitis by inhibiting endogenous miR-21 and let-7b.** Colitis was induced in C57Bl/6 wild-type (WT) mice with 1% Dextran Sulfate Sodium (DSS) diluted in the drinking water from day 0 to 7. Mice (n=6 per group) were orally treated from day 1 to 7 with either PBS (water and 1% DSS groups) or miRNA inhibitors. (A) Body weight. (B) Colon weight on colon length ration. (C) Colonic myeloperoxidase (MPO) amounts. (D-F) Colonic mRNA expression levels of the proinflammatory cytokines TNF (D), IL-6 (E), and IL-1β (F) and the tight junction claudin-2 (G) were measured by RT-qPCR at day 7. For bar plot graphs, values are given as mean ± SEM. Statistical analyses were performed using Kruskal-Wallis followed by Dunn’s post hoc tests. Significant differences were recorded as *p<0.05, **p<0.01, ****p<0.0001.


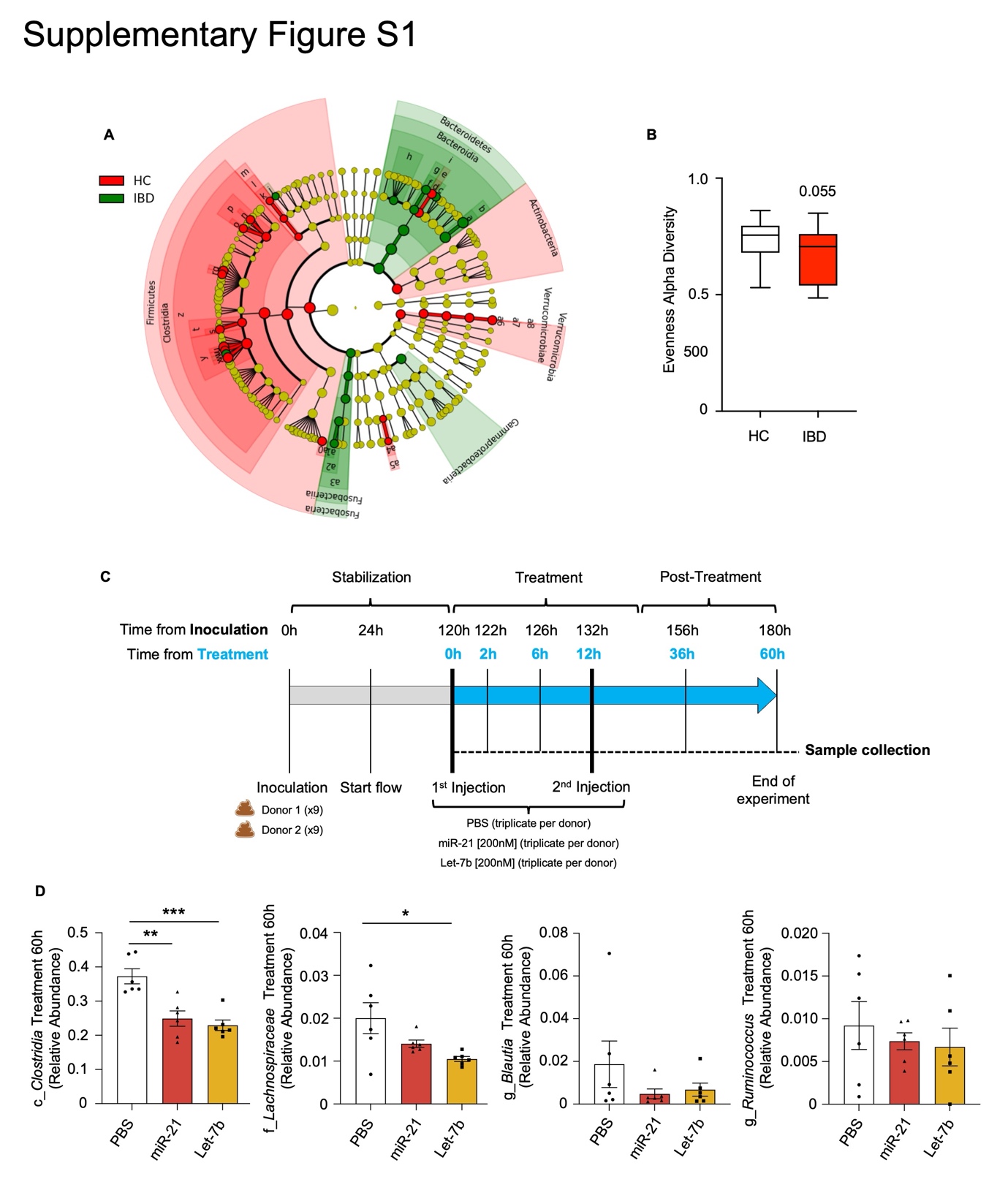


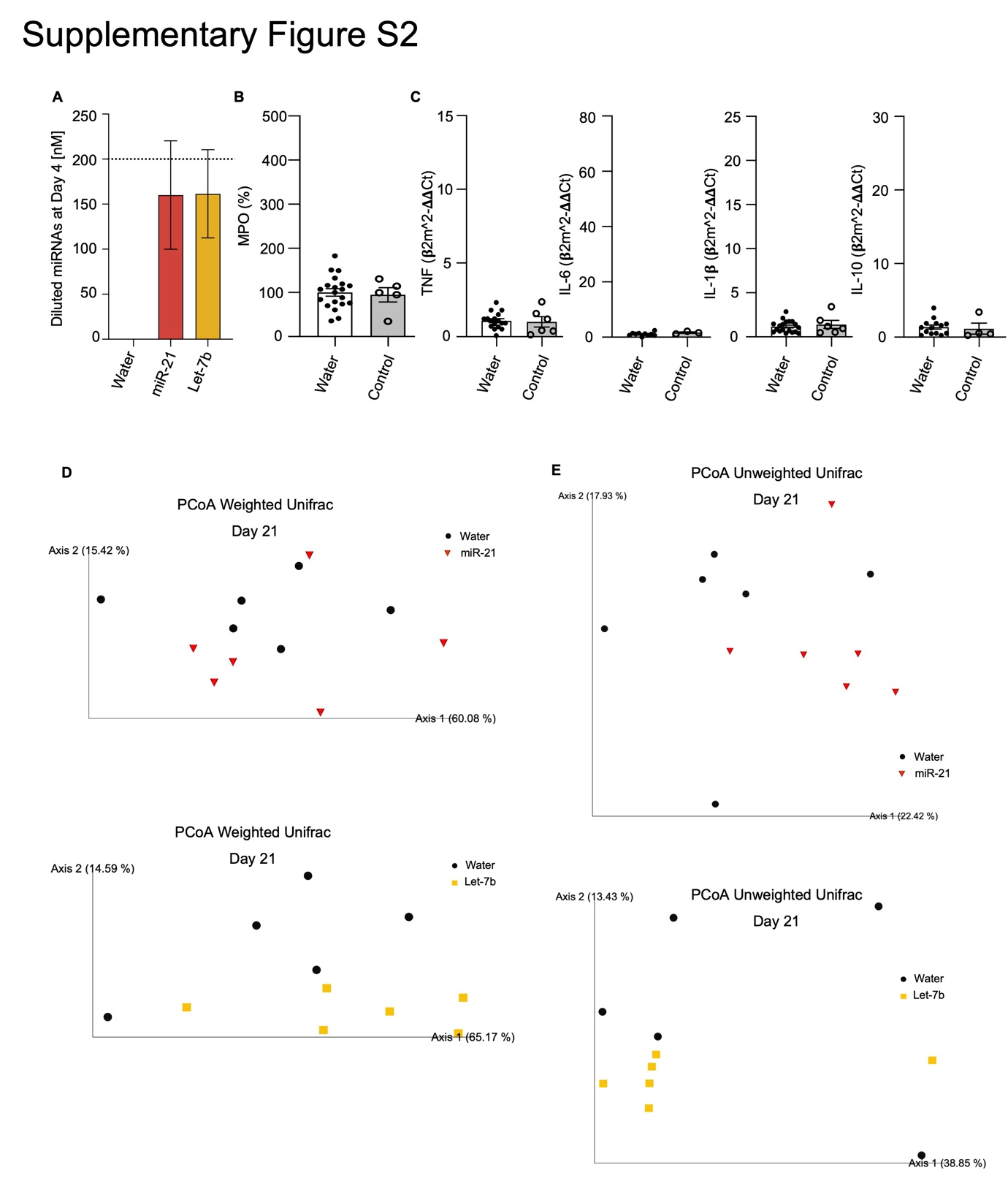


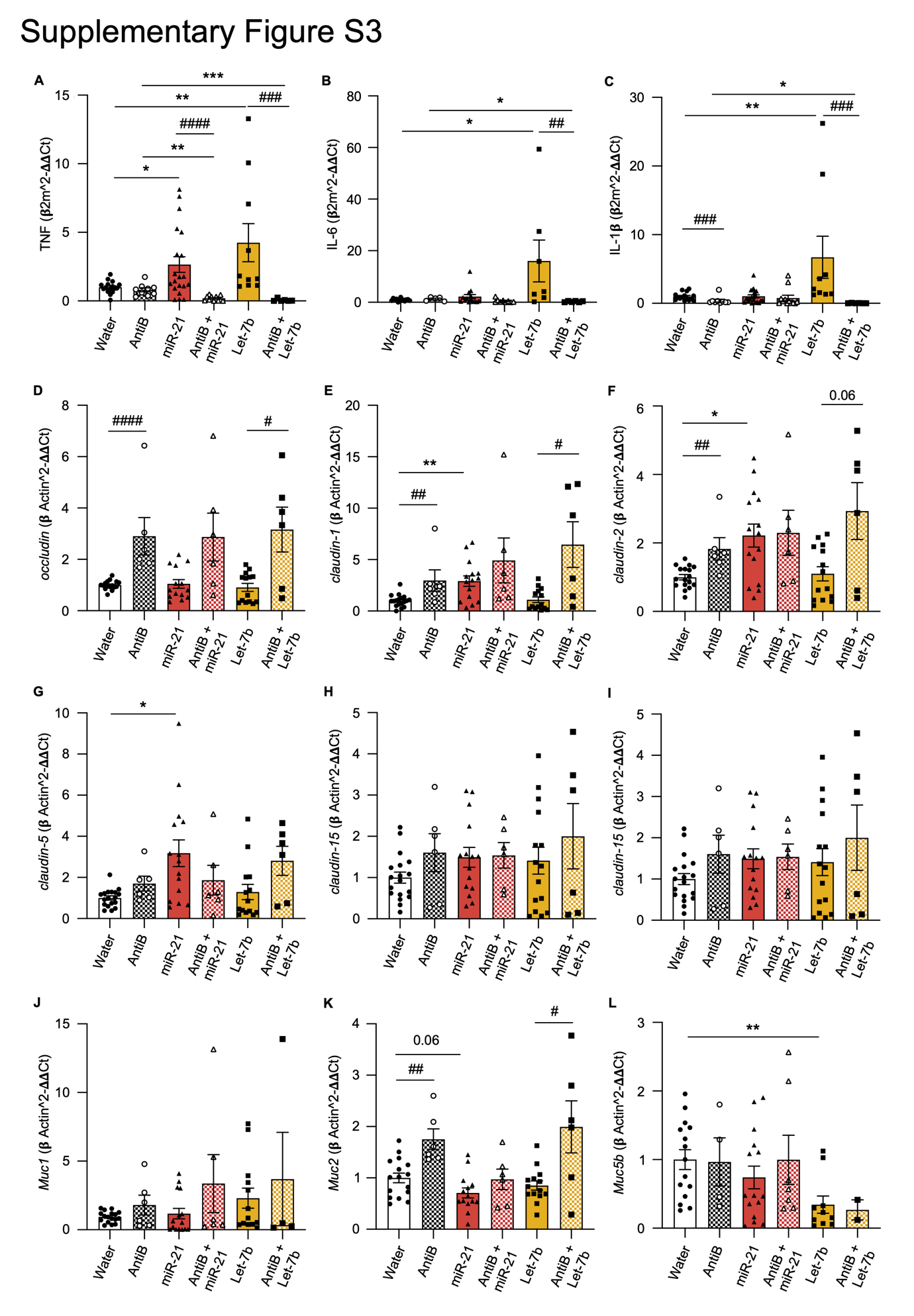


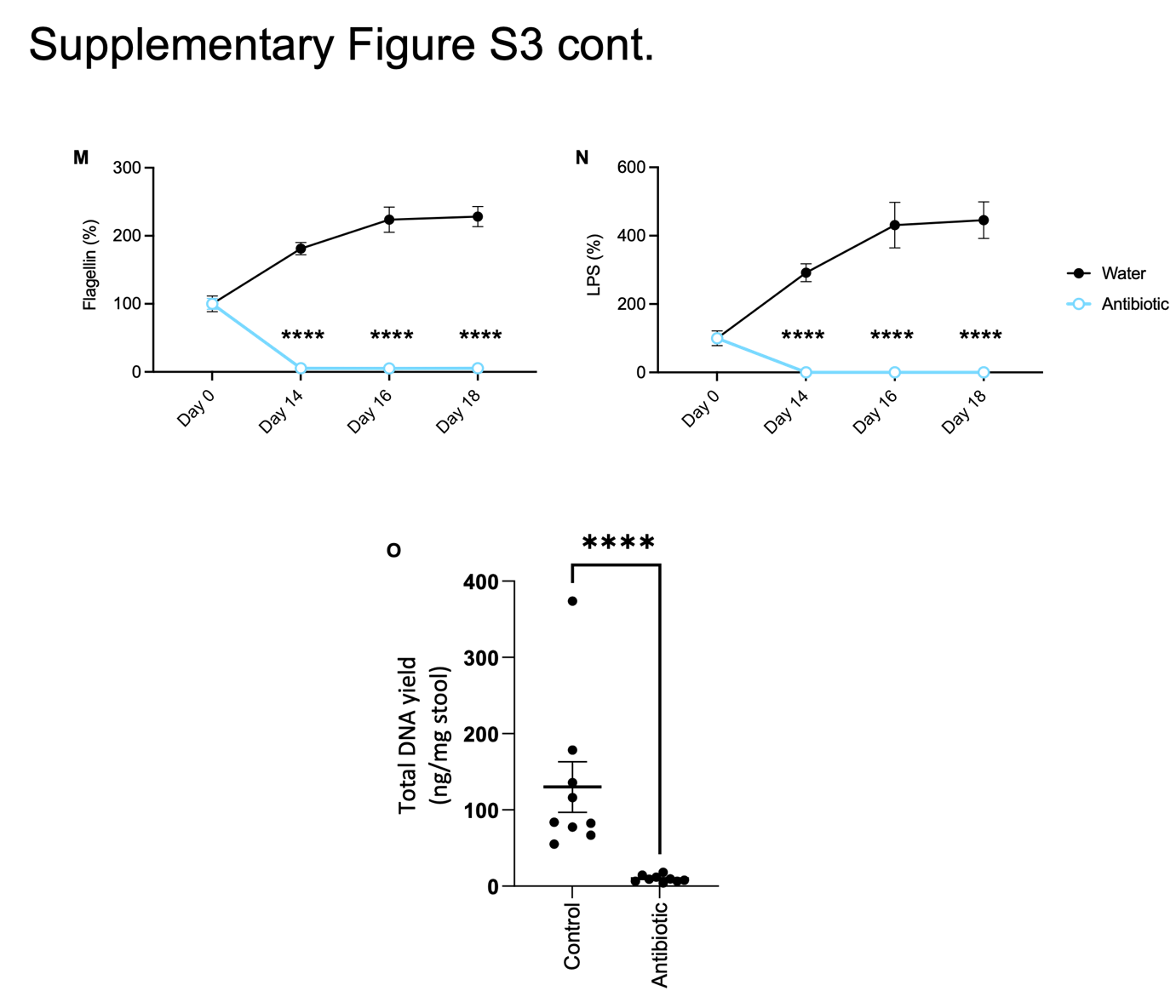


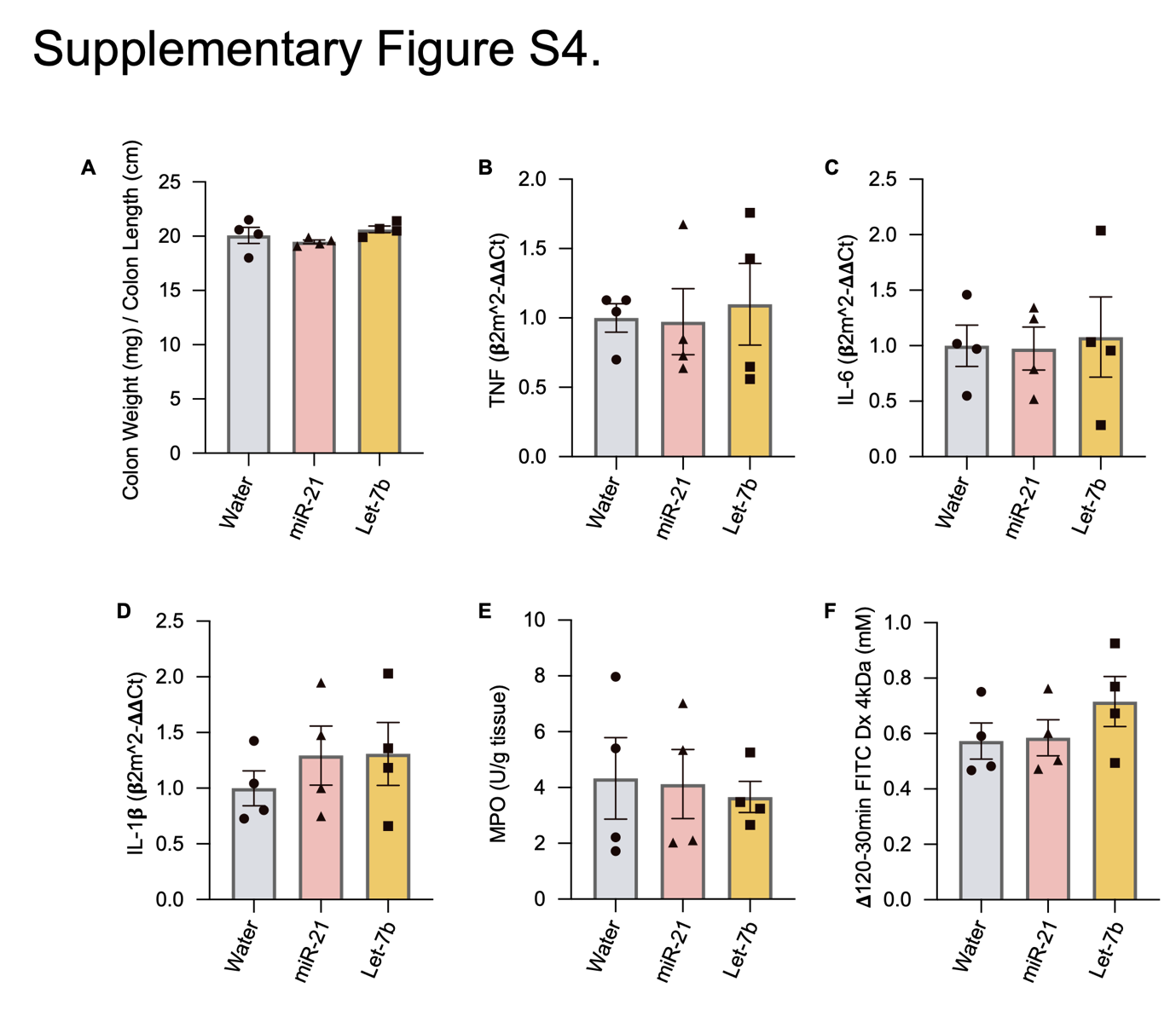


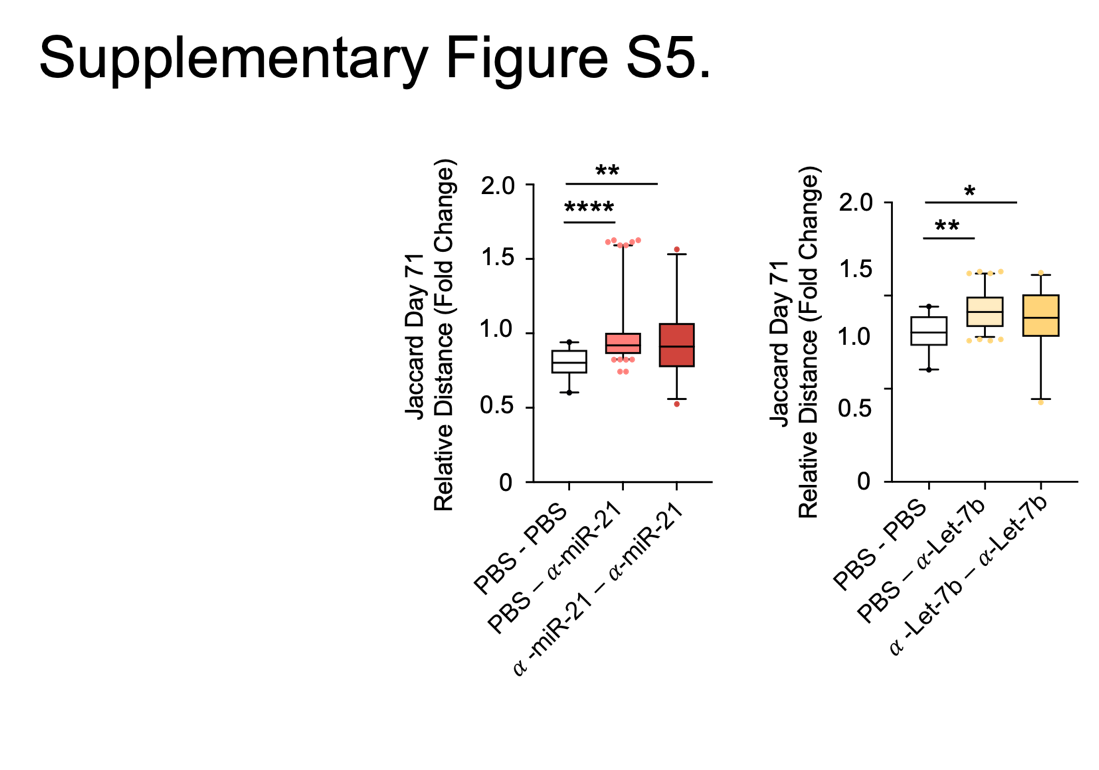


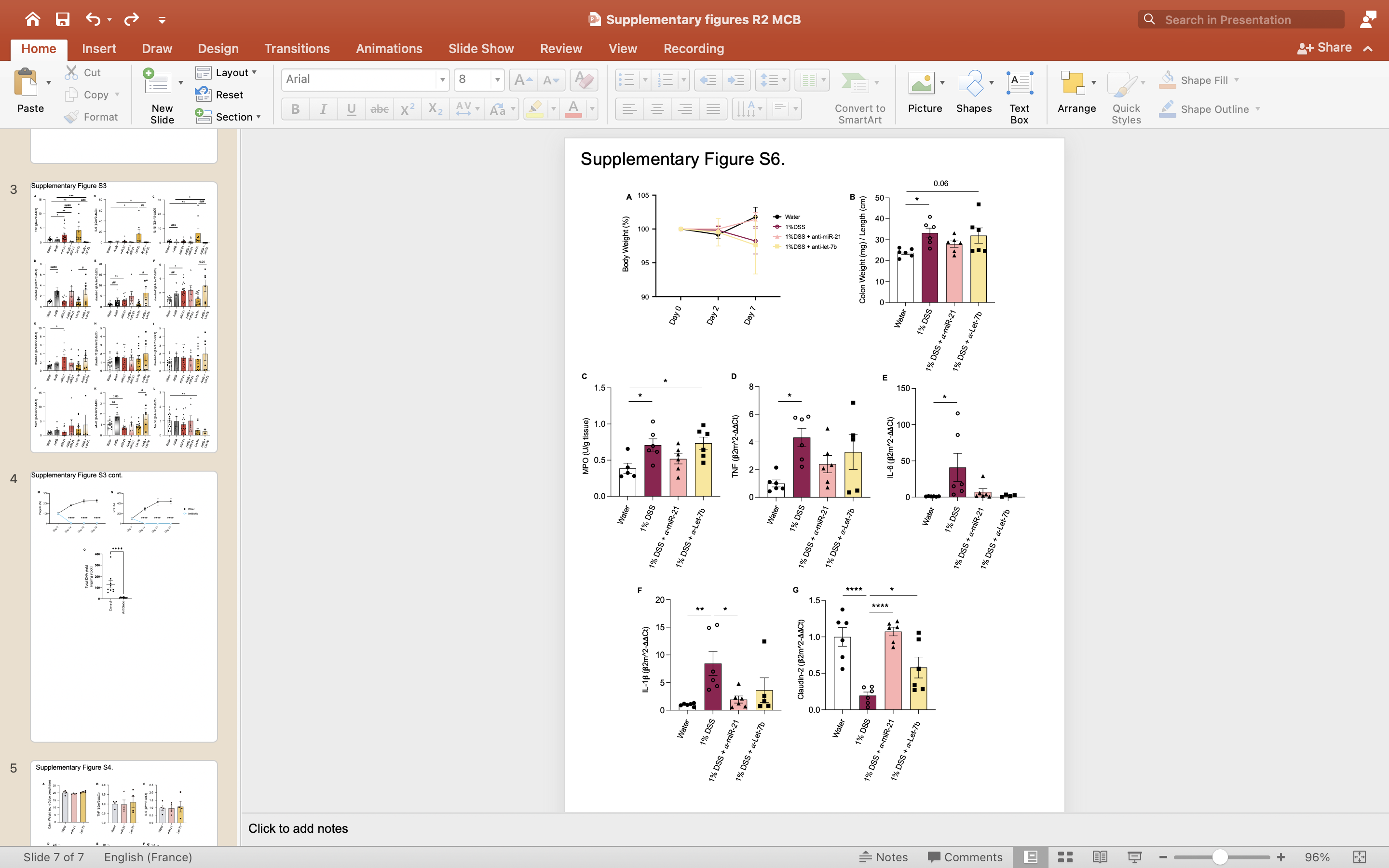

Supplement: Supplemental Material [file KGMI_A_2394249_SM0081.zip › R2 Supplementary file clean.docx]
